# Supplementary material for: Effect of a family-involvement combined aerobic and resistance exercise protocol on cancer-related fatigue in patients with breast cancer during postoperative chemotherapy: study protocol for a quasi-randomised controlled trial
Source: BMJ Open. 2023 Mar 30;13(3):e064850. doi: 10.1136/bmjopen-2022-064850 (PMC10069511; doi:10.1136/bmjopen-2022-064850)
Supplement: Author's manuscript [file bmjopen-2022-064850.draft_revisions.pdf]

BMJ Open

BMJ Open is committed to open peer review. As part of this commitment we make the peer review history of every article we publish publicly available.

When an article is published we post the peer reviewers' comments and the authors' responses online. We also post the versions of the paper that were used during peer review. These are the versions that the peer review comments apply to.

The versions of the paper that follow are the versions that were submitted during the peer review process. They are not the versions of record or the final published versions. They should not be cited or distributed as the published version of this manuscript.

BMJ Open is an open access journal and the full, final, typeset and author-corrected version of record of the manuscript is available on our site with no access controls, subscription charges or pay-per-view fees (http://bmjopen.bmj.com).

If you have any questions on BMJ Open's open peer review process please email info.bmjopen@bmj.com

BMJ Open

Effect of a family-involvement aerobic combined resistance exercise on cancer-related fatigue in patients with Breast Cancer during postoperative chemotherapy:study protocol for a quasi-randomized controlled trial

Journal:	*BMJ Open*
Manuscript ID	bmjopen-2022-064850
Article Type:	Protocol
Date Submitted by the Author:	29-May-2022

Complete List of Authors:	Huang, Chuhan; First Affiliated Hospital of Dalian Medical University, Department of Nursing<br>Cai, Yingjie; First Affiliated Hospital of Dalian Medical University, Department of Nursing; Chiba University, Nursing College<br>Guo, Yufei; First Affiliated Hospital of Dalian Medical University, Department of Nursing<br>Jia, Jingjing; First Affiliated Hospital of Dalian Medical University, Department of Nursing<br>Shi, Tieying; First Affiliated Hospital of Dalian Medical University, Department of Nursing

Keywords:	Breast tumours < ONCOLOGY, SOCIAL MEDICINE, SPORTS MEDICINE

SCHOLARONE™ Manuscripts

Title of the article: Effect of a family-involvement aerobic combined resistance exercise on cancer-related fatigue in patients with Breast Cancer during postoperative chemotherapy: study protocol for a quasi-randomized controlled trial

Full name, postal address and e-mail of the corresponding author:
Tieying Shi, Department of Nursing, The First Affiliated Hospital of Dalian Medical University, Dalian, China , sty11177@163.com

Tel: +86 0411 83635963

Fax: +86 0411 83622844

Tieying Shi is corresponding authors

Full name, department, institution, city and country of all co-authors:
Chuhan Huang,[1] Yingjie Cai,[1,2] Yufei Guo,[1] Jingjing Jia,[1] Tieying Shi,[1]
[1]Department of Nursing, The First Affiliated Hospital of Dalian Medical University, Dalian, China
[2]Nursing College, Chiba University, Chiba, Japan

Keywords:
breast cancer; cancer-related fatigue; postoperative chemotherapy; family-involvement; aerobic combined resistance exercise

Word count, excluding title page, abstract, references, figures and tables:
5622

For peer review only

Effect of a family-involvement aerobic combined resistance exercise on cancer-related fatigue in patients with Breast Cancer during postoperative chemotherapy: study protocol for a quasi-randomized controlled trial

Chuhan Huang,[1] Yingjie Cai,[1,2] Yufei Guo,[1] Jingjing Jia,[1] Tieying Shi,[1]

ABSTRACT

Introduction

Cancer-related fatigue (CRF) is one of the most common and debilitating side effects in patients with breast cancer (BC) throughout postoperative chemotherapy. Family-involvement aerobic combined resistance exercise has been supported as a promising non-pharmacological intervention for the individual symptom relief of cancer-related fatigue, muscle strength, exercise completion, family intimacy and adaptability, and quality of life. However, relevant evidence of using family-involvement aerobic combined resistance exercise for CRF management in patients with BC is lacking report.

Methods and analysis

This study is a quasi-randomized controlled trial involving an 8-week intervention. Seventy patients with BC is recruited from a tertiary medical center in China. The participants from the first oncology department is assigned to the family-involvement aerobic combined resistance exercise group (n=35), while the participants from the second oncology department is assigned to the control group with standard exercise guidance(n=35). The primary outcome is evaluated by the Piper Fatigue Scale-Revised (R-PFS). The secondary outcomes include muscle strength, exercise completion, family intimacy and adaptability, and quality of life, which are evaluated by the stand-up and sit-down chair test, the grip test, the exercise completion rate, the Family Adaptability and Cohesion Scale, Second Edition - Chinese Version (FACESⅡ-CV) and the Functional Assessment of Cancer Therapy - Breast (FACT-B). Analysis of covariance is adopted for comparisons between groups and paired t-test is used for before exercise and after-exercise comparisons within a group.

Key Words breast cancer; cancer-related fatigue; postoperative chemotherapy; family-involvement; aerobic combined resistance exercise

Ethics and dissemination

Ethics approval is obtained from relevant site (PJ-KS-KY-2021-288). The findings of this study is published in a peer-reviewed scientific journal.

Trail registration number

ChiCTR2200055793

Strengths and limitations of this study

For peer review only - http://bmjopen.bmj.com/site/about/guidelines.xhtml

- In this intervention, each participant can choose the type of aerobic exercise they prefer.
- This study will provide exercise instruction videos to increase patients' exercising motivation. An exercise booklet is designed and provided to the participants in both intervention and control groups.
- Given the limited study sites, the sample in this study may only reflect the characteristics of this location .
- Due to the visible nature of the exercise intervention, the blinding of the participants and the investigators cannot be performed in this study which is not a rigorous randomized controlled trial, which might increase the risk of detection bias during the study's implementation.
- The study lacks a long-term follow-up to assess the ongoing effects of family-involvement exercise might be another limitation.

INTRODUCTION

According to the latest data in 2020, the number of new BC patients reached 2.26 million and the number of deaths is 680,000. BC officially replaced lung cancer as the largest cancer worldwide, ranking first in female cancer deaths[1]. In China, there are about 368,000 new cases of BC in 2018, and about 416,000 new cases of BC in 2020[2], showing an increasing trend year by year[3]. The number of cancer survivors has increased, and the 5-year survival rate of BC patients has reached 90%, this has been attributed to the progress made in the realm of screening, diagnosis and therapeutic strategies engaged in breast cancer management[4].

As a common adjuvant therapy after BC surgery, chemotherapy can improve the survival rate and prolong the survival time of BC patients[5,6]. However, patients with low immunity during postoperative chemotherapy are prone to various discomforts, which increase their physical and mental burden and reduce their quality of life. Cancer-related fatigue (CRF) is one of the most common symptoms[7]. According to research reports[8], 80% to 90% of cancer patients will experience cancer-related fatigue during treatment and last for a long time. Cancer-related fatigue not only increases the risk of accidental falls in BC patients but also reduces their tolerance to chemotherapy drugs and increases the incidence of adverse reactions such as nausea and vomiting[9]. In addition, compared with other symptoms such as pain and depression caused by cancer, cancer-related fatigue has a great negative impact on patients' social barriers and social re-employment[10]. Studies have shown that cancer-related fatigue in patients is positively related to muscle strength[11] and health-related quality of life[12]. Therefore, it is necessary to improve cancer-related fatigue in BC patients to promote their return to society and improve their quality of life.

Studies have shown that increasing physical activity in BC patients can improve cancer-related fatigue[13]. Physical activity refers to any physical movement that results in energy

For peer review only

For peer review only - http://bmjopen.bmj.com/site/about/guidelines.xhtml

expenditure caused by skeletal muscle contraction[14], including exercise training such as aerobic exercise, resistance exercise, balance exercise, and flexibility exercise. Among them, aerobic exercise and resistance exercise, as the most common exercise training methods in physical activity, have been proved to be safe and effective[15]. Studies have found that aerobic exercise combined with resistance exercise has the best effect in improving cancer-related fatigue[16].

Although the benefits of aerobic combined resistance exercise for cancer-related fatigue have been proven, BC patients do not exercise well. Good external support is one of the important factors for patients to adhere to exercise[17]. As an important part of external support for patients, family members are the main bearers of daily activities and disease care during hospitalization and after discharge[18]. And studies have shown that, compared with unsupervised exercise training, supervised exercise training with the participation of family members can better ensure the continuous and regular development of exercise, thereby maintaining and improving exercise effects[19, 20]. In addition, increasing disease-related communication between family members and patients can effectively increase patients' confidence in treatment, improve the intimacy and adaptability between patients and their families, and thus improve patients' family functions[21]. Therefore, how to play the active support role of family members in the process of exercise training in BC patients, and then enhance the benefits of exercise in improving cancer-related fatigue in patients is one of the urgent problems to be solved.

The current study, therefore, proposes to assess the feasibility and the preliminary effects of using a family-involvement exercise protocol for alleviating the CRF in patients with BC through a quasi-randomized Controlled Trial(Q-RCT).

METHODS AND MATERIALS

Participant Recruitment and Eligibility Criteria

Patients with postoperative chemotherapy of BC in a Grade A tertiary hospital in Dalian are selected for this study.

The inclusion criteria for patients are: (1) pathological diagnosis of BC; (2) with first postoperative chemotherapy for BC; (3) aged 18 to 65; (4) able to cooperate actively; (5) informed consent to participate in this study voluntarily, and (6) with family members during the intervention for 2 months. The exclusion criteria are: (1) with other serious diseases, such as cardiovascular disease, other cancers, etc.; (2) with exercise contraindications, such as asthma, severe anemia, disc herniation or other diseases; (3) with mental illness, previous mental illness or family medical history.

Each patient corresponds to one family member, the inclusion criteria for family members are: (1) aged above 18, with primary school education or above, and good communication skills; (2) spouse, parents, or children of immediate family; (3) the main caregivers determined by the patient and family members who take care of patients above 2 months; (4) voluntary

For peer review only - http://bmjopen.bmj.com/site/about/guidelines.xhtml

participation in the investigator. The exclusion criteria are: (1) with remuneration; (2) with a previous history of mental illness.

Sample size

According to the previous study[22], cancer-related fatigue score is expected to decrease by 0.6 points. According to the two-sided sample size test formula, a two-sided test with alpha=0.05, and 80% power, this gives a total sample of 30. To account for a 15% attrition rate, the required sample is 70.

Study Design

To avoid contamination among the research subjects, this study adopts a lottery method to randomly divide the hospital's first and second oncology departments into a control group and an intervention group, and select 35 patients in each ward who meet the requirements after screening by the inclusion and exclusion criteria.

The intervention group consist of a sports rehabilitator, a senior clinical nursing specialist, a head nurse, three responsible nurses, and a nursing graduate student. The sports rehabilitator participates in the formulation of the exercise program. The nursing specialist participate in the guidance of the experimental program. The head nurse and three nurses are responsible for quality control, finding problems in the implementation of the program, and making rectifications. While the graduate student is responsible for the intervention of patients and their families, as well as data collection and collation. Group members jointly develope an exercise instruction manual (including exercise forms, exercise methods, exercise precautions, etc.), and record an exercise instruction video.

The researcher explains the purpose and implementation process of the study to the subjects when they are admitted to the hospital for the first chemotherapy (T0). After their agreement to participate, the participants will be required to provide their written informed consent. The participants will be informed that they can withdraw from the study at any moment without any consequences. After obtaining the informed consent of the subjects, the researcher conducts the stand-up and sit-down chair test and the grip test to patients and issues general information questionnaires, R-PFS, FACESⅡ-CV, and FACT-B. The intervention study begins after the patients (and their families) have completed their first assessment.

According to the patient's exercise duration and admission chemotherapy time, the patients are tested with the stand-up and sit-down chair test and the grip test at the time of the second admission chemotherapy (T1) and the fourth admission chemotherapy (T2). The exercise completion rate of the two groups is evaluated, and the researcher issues general information questionnaires, R-PFS, FACESⅡ-CV, and FACT-B to evaluate the effect of exercise intervention. In the process of distributing the questionnaire, if patients could not understand the questionnaire items, the researcher explained it. A Consolidated Standards of Reporting Trials

For peer review only - http://bmjopen.bmj.com/site/about/guidelines.xhtml

flow chart of the study is presented in figure 1. The schedule of trial enrolment, intervention data collection, and assessments are presented in table 1.

Table1 The schedule of tiral enrolment, interventions and assessments

Study period	Beginning of intervention (week 0)	Interxention period (week 4)	End of intervention (week 8)
Inclusion/exclusion criteria	×		
Informed consent	×		
Demograghic characteristics	×		
Randomisation and allocation	×		
R-PFS	×	×	×
The stand-up and sit-down chair test	×	×	×
The grip test	×	×	×
The exercise completion rate		×	×
FACESⅡ-CV	×	×	×
FACT-B	×	×	×

Table1 The schedule of trial enrolment, interventions, and assesments. CRF, cancer-related fatigue; FACESⅡ-CV, the Family Adaptability and Cohesion Scale, Second Edition - Chinese Version; FACT-B, the Functional Assessment of Cancer Therapy - Breast

Control group

When patients are admitted to the hospital for the first chemotherapy, the researcher gives exercise instruction to them, guides patients exercise training through the manual and video, and informs exercise time, form, method, intensity as well as precautions. The patients are followed up monthly to understand their exercise status, and the researcher asks patients why they don't persist in exercising to help them solve their problems, and to encourage them to persist in exercise training.

Intervention group

Based on the control group, the exercise training intervention with family members is presented in Table 2:

Guide family members	Evaluate the patients' life and social environment through family members, and explain to family members the importance of family members accompanying the patient to exercise.
	Distribute exercise manuals to family members and play exercise instruction videos.

For peer review only - http://bmjopen.bmj.com/site/about/guidelines.xhtml

	Instruct family members to master the exercise time, method, exercise standard posture, exercise precautions, and precautions for the use of elastic bands and dumbbells for BC patients.	
Supervise family members	Issue supervision diaries to family members[1].	Remind and supervise family members to fill in the supervision diary through WeChat or phone every month.
		When the patient is readmitted to the hospital, he is required to carry the supervision diary to check the record in the diary.
	Supervise family members via WeChat.	Establish a special WeChat group for family members, send exercise-related health knowledge to the WeChat group every week, and regularly remind family members to urge patients to exercise.
		Encourage family members to ask questions actively in the WeChat group and provide timely feedback on the supervision situation and feelings, to answer relevant questions for family members in the process of guiding and supervising patients.
		Appropriate praise and rewards are given to the family members who first complete the monitoring goals every week, forming a competition mechanism and stimulating interest.
Follow up with family members	Make phone calls or face-to-face follow-up with family member monthly, ask them the questions they encountered during the care of the patients and answer them.	
	Ask the patient about the training situation. If patients cannot insist on exercise training, further inquire and analyze the reasons why patients cannot insist, and help the family to solve the problem.	
Family members accompany patients	Participate in the whole process of the patients' exercise and provide the patients with full emotional support. Before exercising, choose the way of aerobic exercise together with the patients according to the patient's preference and play music for patients to relax while they exercise.	
Family members supervise patients	Give exercise guidance to the patient. Guide the patients in the correct exercise method by playing the video. When the patients make an error or non-standard movement during the exercise, correct it in time.	
	Supervise the patient exercising. Take photos or videos while the	

For peer review only - http://bmjopen.bmj.com/site/about/guidelines.xhtml

	patients are moving to record the patients' movement. Keep a supervision diary after each patient's exercise and upload exercise photos or videos in the WeChat group to ensure the effect of family supervision.
Family members encourage patients	Encourage and support the patients and enhance their confidence; encourage the patients to tell themselves about the difficulties encountered during exercise, and tell them that they will overcome it together.

[1]The first part of the supervision diary is basic information, including initials of family members, initials of patients, the relationship between family members and patients, height, weight, initials of the doctor in charge, date of chemotherapy, etc. This part is filled out by the researcher and distributed to the family members. The second part includes the exercise date, time, location, exercise duration, frequency and number of groups, the patient's exercise status, and whether he or she participated in the exercise guidance and supervision throughout the exercise.

Table 2 The exercise training intervention with family members

Aerobic combined resistance exercise

The exercise training lasts for 8 weeks, and the patients can select the most comfortable period of the week according to the patients' situation every week. Each training time is scheduled for half an hour after a meal, and avoid training on an empty stomach or a full meal. Each workout includes aerobic and resistance exercises which both are progressive. It is allowed 5 minutes of rest between aerobic and resistance exercise. Aerobic exercise is accomplished by cycling, treadmill, or brisk walking on the ground, depending on the patients' preference: twice a week for 15 to 20 minutes in week1-4; three times a week for 25 to 35 minutes in week 5-8. Resistance exercise used 0.45kg dumbbells or a mineral water bottle filled with 500ml of water and a 1.8cm brown elastic band named TheraBand and provided by the researchers as the main exercise tools, including four resistance exercise movements (hip abduction, seated knee raising, raise before standing, standing lateral raise): twice a week in week 1-4, two sets of each exercise, each set of 8-12 times; three times a week in week 5-8, three sets of each exercise, each set of 8-12 times, with plenty of rest between sets[23-26].

Hip abduction: When preparing, the body is in a standing position, the ankle of one foot is covered with the elastic band, the other foot is on the elastic band, the opposite hand holds the seat, and the supporting foot firmly grasps the ground to keep the trunk stable; at the beginning The buttocks exert force and pull the elastic band to the side until the angle between the practice leg and the supporting foot is about 30 degrees, and pause for about one second, then slowly restore, alternating with the left and right feet.

Seated knee raising: When preparing, sit in a sitting position with a straight back, hold the seat with both hands, keep the upper torso upright, step on one end of the elastic band with one foot, and put the ankle of one foot on the elastic band to bear the weight; lift the knee at the beginning To the maximum angle of joint activity, and pause for about one second, then slowly lower, alternating left and right.

For peer review only - http://bmjopen.bmj.com/site/about/guidelines.xhtml

Raise before standing: Stand with feet parallel, head straight and look straight ahead, hold dumbbells at both sides of the body with both hands, raise arms in front of the body, pause for about one second, and then slowly restore. Inhale during the horizontal lift, exhale when returning to the original position.

Standing lateral raise: Stand with feet parallel, head straight, look straight ahead, hold dumbbells on both sides of the body, raise arms by the side, bend the elbows slightly, focus on the shoulders, and focus on the shoulders. When the arm is raised to a horizontal position, pause for about one second, slowly lower it, and restore the arm to the initial position. Inhale during the horizontal lift, exhale when returning to the original position.

The intensity of aerobic exercise should be moderate, that is, the Borg subjective fatigue scale score should reach 13-14 points (feeling a little hard); the intensity of resistance exercise should be tolerated by the patient. If there is obvious fatigue and muscle pain during or after training, the intensity of physical activity should be appropriately reduced. If patients have severe pain, discomfort and other symptoms occur, they should immediately stop the activity and be admitted to the hospital for re-examination[27,28].

During the exercise, pay attention to observe the patients' reaction and adjust the elastic band or exercise speed and intensity in time; pay attention to the patients' exercise force should not be too large to avoid stretching the wound or the place where the catheter is placed; pay attention to the patient's movement at any time, if the patients are dizzy, discomfort and other adverse events occur, they should be promptly instructed to stop exercising and visit the hospital if necessary.

Outcome measurements

The outcome measurements for this pilot will include two categories, namely, baseline assessments and clinical outcomes.

Demographic and clinical characteristics of the participants

A self-designed demographic and clinical data form is employed to collect the participants' sociodemographic data (eg, age, education background, employment status, marital status, and household income), the participants' medical history (eg, date of diagnosis, the current stage of BC and date and type of treatment) and family members' sociodemographic data at baseline (T0).

Primary outcomes: cancer-related fatigue

The Piper Fatigue Scale-Revised (R-PFS)[29]: The R-PFS is adopted to assess the participants' subjective fatigue. This self-administered questionnaire contains 22 items whose scores range from 0 to 10 and includes four domains of subjective fatigue. The R-PFS is available in a simplified Chinese version[30], with high reliability and validity and has high reliability in the BC population.

Secondary outcomes: muscle power, exercise completion, family intimacy and adaptability and QOL

For peer review only - http://bmjopen.bmj.com/site/about/guidelines.xhtml

The muscle strength exercise completion, family intimacy and adaptability, and QoL of the patients with BC as the secondary outcomes are measured at T0, T1, and T2 using the stand-up and sit-down chair test, the grip test, the exercise completion rate, the FACESⅡ-CV and the FACT-B.

1. The stand-up and sit-down chair test (number of times standing up from the chair within 30 s) [31]: This test is used to evaluate the leg strength of the subject. The procedure of testing: 1) Put an upright chair (or a folding chair) against the wall (for the sake of safety). 2) The subject sits in the middle of the chair with the right foot and the left foot apart equal to the shoulders, and one foot may be put slightly front and the other slightly back. Both arms are crossed around the waist and close to the chest. 3) During the test, the subject should completely stand up and then completely sit down. 4) Record the number of times that the subject stands up from and sits down in the chair within 30 s. 5) For safety purposes or when necessary, the subject may use her arms for assistance.

2. The grip test[28]: This test is used to evaluate the arm strength of the subject. Use domestic CAMRY (model EH101) electronic grip strength meter, this grip strength meter is in kg/Ib, the maximum range is 90kg/198Ib, and the division value is 0.1kg/0.2Ib. The subjects are in a standing position, with their hands hanging down naturally, their feet are flush with both shoulders, and the hands are not close to the body.

3. Exercise completion rate: This test is used to assess the performance of the two groups of exercise. The exercise completion rate in this study = the number of subjects in the intervention group who completed 18 or more training sessions (over 90% of the total training volume) ÷ the total number of people in the intervention group × 100%.

4. The Family Adaptability and Cohesion Scale, Second Edition - Chinese Version (FACESⅡ-CV)[32]: The FACESⅡ-CV is adopted to assess the participants' family intimacy and adaptability. A higher score demonstrates higher intimacy and adaptability. The FACESⅡ-CV is available in a simplified Chinese version, with high reliability and validity.

5. The Functional Assessment of Cancer Therapy - Breast (FACT-B)[33]: The FACT-B is adopted to assess the participants' QoL. A higher score demonstrates better QoL. The FACT-B is available in a simplified Chinese version, with adequate psychometric properties reported among patients with BC.

Data analysis

The study results underwent statistical analysis with SPSS software version 25.0. Analysis of covariance is adopted for comparisons between groups and paired t-test is used for before exercise and after-exercise comparisons within a group, whereas $p < 0.05$ represents there is a significant difference between the test results.

Data management

For peer review only - http://bmjopen.bmj.com/site/about/guidelines.xhtml

After data collection is complete, all paper data is converted to electronic data. All data is independently recorded by two researchers using Excel. The software automatically checks inconsistent or problematic data based on the inspection results and generates a data problem table. After all, data have been confirmed, reconciled, and stored in an electronic database, participants' identifying information (eg, real name) will not appear in the relevant reports of the trial to protect their privacy. Only researchers directly involved in the analysis of this study have access to the final trial dataset, which contains only coded data.

Data sharing statement

Technical appendix, statistical codes and dataset will be available at any time by contacting the corresponding author.

Patient and public involvement

Patients and/or public were not involved in designing this study.

Ethics and dissemination

Ethical approval is granted by the Ethics Committee of the First Affiliated Hospital of Dalian Medical University (PJ-KS-KY-2021-288) and is retrospectively registered in the Chinese Clinical Trial Registry (registration number: ChiCTR2200055793). Only the participants who signed the informed consent document are included in the study.

DISCUSSION

As one of the most common symptom clusters in patients with BC, the CRF can significantly deteriorate patients' QoL and daily functioning[34]. An increasing number of studies have demonstrated that aerobic combined resistance exercise has beneficial effects on symptom management in patients with cancer[35]; however, due to the time and intensity of exercise and individual differences, the effect of each study varies. Kajal Gokal[36] conducted a home-based walking intervention based on self-management in 25 BC patients receiving chemotherapy. The results showed that self-management exercise intervention can improve patients' fatigue symptoms and improve their physical activity level. However, some patients are difficult to adhere to exercise training, causing the patients to stop exercising. Kyeong[37] conducted a 12-week randomized controlled trial on 356 BC patients. The results showed that aerobic combined resistance exercise can improve patients' fatigue symptoms, muscle strength, and quality of life, but because some patients cannot persist in exercise, the advantages of exercise over traditional exercise programs are not obvious. Existing studies have confirmed that family-involvement exercise interventions have many benefits in stroke[38], coronary heart disease[39], lung cancer[40], and other patients, while there are few reports on the effect of family-involvement exercise interventions on BC patients in China. This highlights a great need to explore the effects of family-involvement exercise on the CRF in patients with BC.

For peer review only - http://bmjopen.bmj.com/site/about/guidelines.xhtml

The principal strength (and novel aspect) of this study resides in the fact that in the intervention, each participant chooses the type of aerobic exercise they prefer. And the study provides exercise instruction videos to increase patients' exercising motivation. In addition, we aim to provide new evidence on the cost-effectiveness of this kind of intervention (to our knowledge, virtually no evidence has previously been reported in this regard). Moreover, an exercise booklet is designed and provided to the participants in both the intervention and control groups. The information listed in this booklet is comprehensively adapted from relevant national guidelines, professional bodies, and research evidence in published peer-reviewed articles.

This study also has some limitations. Given the limited study sites, the study sample in this study may not offer a completely representative sample of patients with BC. Due to the visible nature of the exercise intervention, the blinding of the participants and the investigators cannot be performed in this study, which might increase the risk of detection bias during the study's implementation. The lack of long-term follow-up to assess the ongoing effects of family-involvement exercise might be another limitation, but this can be considered in the future full-scale trial as one of the main study outcomes. Furthermore, this study is not a rigorous randomized controlled trial, and a future multicentre large-scale main RCT should be conducted in the future to further summarize the research evidence on the effects of family-involvement exercise on the management of patients with CPF.

This study will use a quasi-randomized controlled trial to assess the feasibility and preliminary effects of a family-involvement exercise program for alleviating the CRF in patients with BC under postoperative chemotherapy. The convenience of the family-involvement exercise for the management of the CRF may provide patients with BC, healthcare professionals, and policy-makers with further guidance in CRF management in the long run.

Author affiliations

[1]Department of Nursing, The First Affiliated Hospital of Dalian Medical University, Dalian, China

[2]Nursing College, Chiba University, Chiba, Japan

Contributorship statement Chuhan Huang conceived and designed the study, and Tieying Shi oversaw the research team and research process. Yingjie Cai and Chuhan Huang were the main implementers of the study and drafted the manuscript. Jingjing Jia wrote the ethics review confirmation. Yufei Guo participated in the design of the study and assisted in drafting the manuscript. All authors have read and agree to the final version of the manuscript.

Funding The authors have not declared a specific grant for this research from any funding agency in the public, commercial or not-for-profit sectors.

Competing interests None declared.

Patient consent for publication Not required.

For peer review only - http://bmjopen.bmj.com/site/about/guidelines.xhtml

Provenance and peer review Not commissioned; externally peer reviewed.

Ethics approval The study has been approved by the Ethics Committee of the First Affiliated Hospital of Dalian Medical University, China(PJ-KS-KY-2021-288).

REFERENCES:

[1]Sung H,Global cancer statistics 2020: GLOBOCAN estimates of incidence and mortality world wide for 36 cancers in 185 countries[J]. A Cancer Journal for Clinicians. 2021, 71(3): 209-249

[2]World Health Organization. Global Cancer Observatory(GCO):Cancer Today[EB/OL] https://gcoiarcfr/today/home, 2021-02-03.

[3]Chen W, Zheng R, Baade PD, et al. Cancer statistics in China, 2015[J]. A Cancer Journal for Clinicians. 2016 ,66(2):115-32.

[4]Li T, Mello-Thoms C, Brennan PC. Descriptive epidemiology of breast cancer in China: incidence, mortality, survival and prevalence[J]. Breast Cancer Research and Treatment, 2016, 159(3): 395-406.

[5]Breast Cancer Professional Committee of China Anti-Cancer Association. Chinese Anti-Cancer Association Guidelines and Standards for Breast Cancer Diagnosis and Treatment (2021 edition)[J].China Oncology, 2021, 31(10): 954-1040.

[6]Peng Z, Wei J, Lu X, et al.Diagnosis and treatment pattern among rural and urban breast cancer patients in Southwest China from 2005 to 2009[J]. Oncotarget. 2016 , 7(47): 78168-78179.

[7]Sue Kim, Jeehee Han, Min Young Lee, et al. The experience of cancer-related fatigue, exercise and exercise adherence among women breast cancer survivors: Insights from focus group interviews[J]. Journal of Clinical Nursing, 2020, 29(5-6): 758-769.

[8]Fabi A, Bhargava R, Fatigoni S, et al. Cancer-related Fatigue: Esmo Clinical Practice Guidelines for Diagnosis and Treatment[J]. Annals of Oncology, 2020, 31(6): 713-723.

[9]Abrahams HJG, Gielissen MFM, Schmits IC, et al. Risk factors, prevalence, and course of severe fatigue after breast cancer treatment: a meta-analysis involving 12 327 breast cancer survivors[J]. Annals of Oncology, 2016, 27(6): 965-974.

[10]Ekenga CC, Perez M, Margenthaler JA, et al. Early-stage Breast Cancer and Employment Participation after 2 Years of Follow-up: A Comparison with Age-matched Controls[J]. Cancer, 2018, 124(9): 2026-2035.

[11]Mustian KM, Alfano CM, Heckler CE, et al. Comparison of pharmaceutical, psychological, and exercise treatments for cancer-related fatigue: a meta-analysis[J]. JAMA Oncology, 2017, 3(7):961-968.

[12]Ehlers Diane K, DuBois Kelli, Salerno Elizabeth A. The effects of exercise on cancer-related fatigue in breast cancer patients during primary treatment: a meta-analysis and systematic review[J]. Expert Review of Anticancer Therapy, 2020, 20(10): 865-877.

[13]Lahart Ian M, Metsios George S, Nevill Alan M, et al. Physical activity for women with breast cancer after adjuvant therapy[J]. The Cochrane database of systematic reviews, 2018, 1(1): CD011292.

[14]Caspersen CJ, Powell KE, Christenson GM. Physical activity, exercise, and physical fitness: definitions and distinctions for health-related research[J]. Public Health Reports, 1985, 100(2): 126-131.

[15]Joel T Fuller, Michael C Hartland, Luke T Maloney, et al. Therapeutic effects of aerobic and

For peer review only - http://bmjopen.bmj.com/site/about/guidelines.xhtml

resistance exercises for cancer survivors: a systematic review of meta-analyses of clinical trials[J]. British Journal of Sports Medicine,2018, 52(20): 1311.
[16]ZHOU Shuai, JIANG Jinfang, ZHANG Ling, et al. Different Exercise Therapies in Patients with Cancer-related Fatigue: A Network Meta-Analysis[J].Nursing Journal of Chinese People's Liberation Army, 2021, 38(08): 65-68+88.
[17]DENG Yangqing, PAN Cen, WANG Qinglan,et al.Qualitative Research on Exercise Experience of Patients with Breast Cancer: A Meta-synthesis[J].Journal of Nursing(China), 2021, 28(13): 34-39.
[18]Liu Min, Li Guohong, Xue Xiaoming, et al. Effect of family-participated continuation nursing model on the quality of life of discharged patients with colorectal cancer[J].Chinese Journal of Gerontology, 2020, 40(23): 5080-5085.
[19]Wanderson Divino Nilo dos Santos, Amilton Vieira, Claudio Andre Barbosa de Lira, et al. Once a Week Resistance Training Improves Muscular Strength in Breast Cancer Survivors: A Randomized Controlled Trial[J]. Integrative Cancer Therapies,2019,18, 1534735419879748..
[20]Witlox L, Velthuis MJ, Boer JH, et al. Attendance and compliance with an exercise program during localized breast cancer treatment in a randomized controlled trial: The PACT study[J]. PLoS One,2019, 14(5): e0215517.
[21]YUN Qingping, JI Ying, JIANG Xuewen, et al.Path analysis of health behavior improvement in chronic disease patients based on family function theory[J].Chinese Journal of Disease Control & Prevention, 2019, 23(07): 861-865.
[22]Zhu Hongyu, Zhang Yunfei, Ke Xi, et al. The effect of graded walking exercise on cancer-related fatigue in patients with gastric cancer during chemo therapy[J].Chinese Journal of Nursing, 2021, 56(05): 652-658.
[23]Anne Marthe Schreuder, Anne M. Eskes, Rosanna G.M. van Langen, et al. Active involvement of family members in postoperative care after esophageal or pancreatic resection: A feasibility study[J]. Surgery, 2019, 166(5): 769-777.
[24]Huang HP, Wen FH, Yang TY, et al. The effect of a 12-week home-based walking program on reducing fatigue in women with breast cancer undergoing chemotherapy: A randomized controlled study[J]. International Journal of Nursing Studies, 2019, 11(9): 103376.
[25]Li He, Wang Yixiao, Shi Tieying,et al. Effect of Progressive Resistance Training on Upper Limb Function in Breast Cancer Patients[J].Chinese Journal of Rehabilitation Theory and Practice, 2015, 21(11): 1356-1359.
[26]Qin Bo. Effects of elastic band resistance training on lower limb muscle strength and physical activity in the elderly[D].Shanghai Normal University, 2021.
[27]Dong Xin, Mo Yihan, WANG Xiuhua. Effects of resistance exercise on muscle mass, muscle strength and physical performance in the elderly at risk of sarcopenia[J].Chinese Nursing Management, 2021, 21(08): 1190-1195.
[28]Li He. Postoperative progressive resistance training on the rehabilitation of breast cancer patients[D]. Dalian Medical University, 2015.
[29]So WKW, Dodgson J, Tai JWM. Fatigue and quality of life among Chinese patients with hematologic malignancy after bone marrow transplantation[J]. Cancer Nursing, 2003, 26(3):211-219.
[30]Li L, Zhu LH, Cao MQ. Progress in the diagnosis and evaluation scale of cancer-related fatigue[J]. Xinjiang Medcine Journal, 2016, 46(11): 1458-1461,1465.
[31]Xiaosheng Dong, Xiangren Yi, Dezong Gao, et al. The effects of the combined exercise intervention based on internet and social media software (CEIBISMS) on quality of life, muscle

For peer review only - http://bmjopen.bmj.com/site/about/guidelines.xhtml

strength and cardiorespiratory capacity in Chinese postoperative breast cancer patients:a randomized controlled trial[J]. BioMed Central,2019,17(1): 109.
[32]Fei Lipeng, Shen Qijie, Zheng Yanping, et al. Preliminary evaluation of “Family Intimacy and Adaptability Scale” and “Family Environment Scale”[J].Chinese Mental Health Journal, 1991(05): 198-202+238.
[33]Wan Chonghua, Tang Xueliang. Introduction to the Chinese version of FACT-B, a measure of quality of life for breast cancer patients[J]. China Cancer, 2002, 11(6):318-320.
[34]Ruiz-Casado A, Álvarez-Bustos A, de Pedro CG, et al. Cancer-related Fatigue in Breast Cancer Survivors: A Review[J]. Clinical Breast Cancer. 2021, 21(1): 10-25.
[35]BOLAM K A, MIJWEL S, RUNDQVIST H, et al. Two-year follow-up of the OptiTrain randomised controlled exercise trial[J]. Breast Cancer Research and Treatment, 2019, 175(3): 637-648.
[36]Gokal Kajal, Wallis Deborah, Ahmed Samreen, et al. Effects of a self-managed home-based walking intervention on psychosocial health outcomes for breast cancer patients receiving chemotherapy: a randomised controlled trial[J]. Supportive care in cancer, 2016, 24(3): 1139-66..
[37]Kyeong Eun Uhm, Ji Sung Yoo, Seung Hyun Chung, et al. Effects of exercise intervention in breast cancer patients: is mobile health (mHealth) with pedometer more effective than conventional program using brochure[J]. Breast Cancer Research and Treatment,2017,161(3):443-452.
[38]Galvin R, Cusack T, Stokes E. A randomised controlled trial evaluating family mediated exercise (FAME) therapy following stroke[J]. BMC Neurology. 2008, 20(8): 22.
[39]Ma Lili, Sun Suping, YAN Xueqing, et al. Effects of cardiac rehabilitation with the participation of spouses on the patients undergoing PCI and their spouses[J].Chinese Nursing Management, 2020, 20(03): 393-398.
[40]Kelly J. Lafaro, Dan J. Raz, Jae Y. Kim, et al. Pilot study of a telehealth perioperative physical activity intervention for older adults with cancer and their caregivers[J]. Supportive Care in Cancer,2020,28(8): 3867-3876.

Figure 1 A Consolidated Standards of Reporting Trials flow chart of the study

For peer review only

For peer review only - http://bmjopen.bmj.com/site/about/guidelines.xhtml

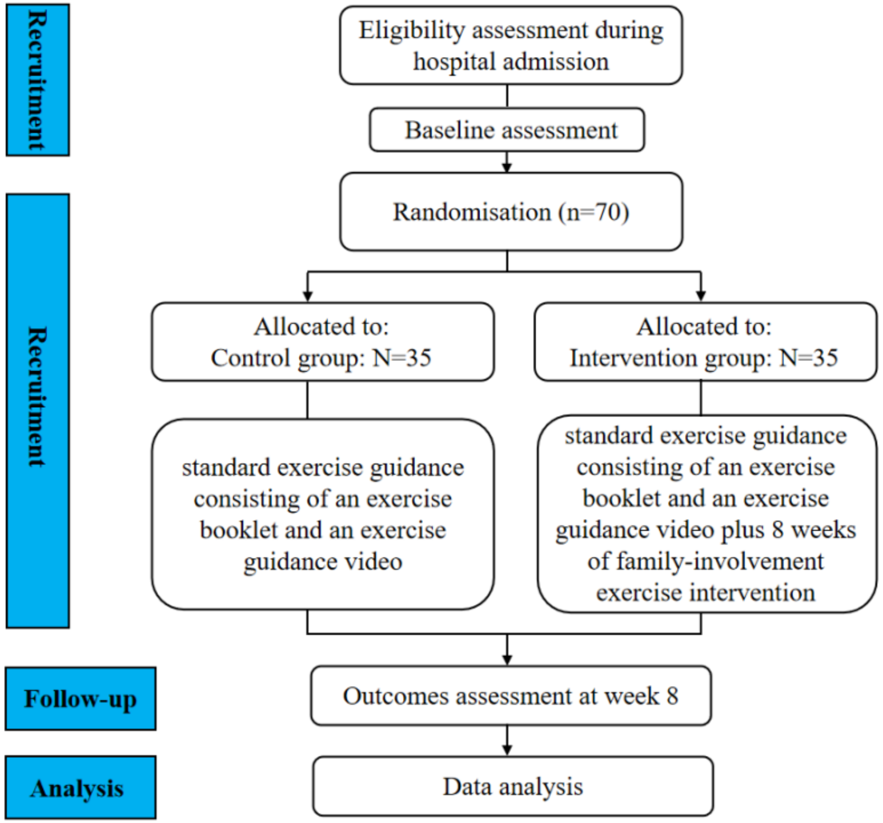
Recruitment
Eligibility assessment during hospital admission
Baseline assessment
Randomisation (n=70)
Recruitment
Allocated to:
Control group: N=35
Allocated to:
Intervention group: N=35
standard exercise guidance consisting of an exercise booklet and an exercise guidance video
standard exercise guidance consisting of an exercise booklet and an exercise guidance video plus 8 weeks of family-involvement exercise intervention
Follow-up
Outcomes assessment at week 8
Analysis
Data analysis

CONSORT 2010 checklist of information to include when reporting a randomised trial*

Section/Topic	Item No	Checklist item	Reported on page No
**Title and abstract**			
	1a	Identification as a randomised trial in the title	No.1
	1b	Structured summary of trial design, methods, results, and conclusions (for specific guidance see CONSORT for abstracts)	No.1
**Introduction**			
Background and objectives	2a	Scientific background and explanation of rationale	No.1-2
	2b	Specific objectives or hypotheses	No.2
**Methods**			
Trial design	3a	Description of trial design (such as parallel, factorial) including allocation ratio	No.4
	3b	Important changes to methods after trial commencement (such as eligibility criteria), with reasons	No.4
Participants	4a	Eligibility criteria for participants	No.3
	4b	Settings and locations where the data were collected	No.3-4
Interventions	5	The interventions for each group with sufficient details to allow replication, including how and when they were actually administered	No.6-9
Outcomes	6a	Completely defined pre-specified primary and secondary outcome measures, including how and when they were assessed	No.9
	6b	Any changes to trial outcomes after the trial commenced, with reasons	Not
Sample size	7a	How sample size was determined	No.4
	7b	When applicable, explanation of any interim analyses and stopping guidelines	
Randomisation:			
Sequence generation	8a	Method used to generate the random allocation sequence	Not
	8b	Type of randomisation; details of any restriction (such as blocking and block size)	No.4
Allocation concealment mechanism	9	Mechanism used to implement the random allocation sequence (such as sequentially numbered containers), describing any steps taken to conceal the sequence until interventions were assigned	No.4
Implementation	10	Who generated the random allocation sequence, who enrolled participants, and who assigned participants to interventions	No.4
Blinding	11a	If done, who was blinded after assignment to interventions (for example, participants, care providers, those	Not

For peer review only - http://bmjopen.bmj.com/site/about/guidelines.xhtml

		assessing outcomes) and how	
	11b	If relevant, description of the similarity of interventions	Not
Statistical methods	12a	Statistical methods used to compare groups for primary and secondary outcomes	No.10
	12b	Methods for additional analyses, such as subgroup analyses and adjusted analyses	
**Results**			
Participant flow (a diagram is strongly recommended)	13a	For each group, the numbers of participants who were randomly assigned, received intended treatment, and were analysed for the primary outcome	Not
	13b	For each group, losses and exclusions after randomisation, together with reasons	Not
Recruitment	14a	Dates defining the periods of recruitment and follow-up	Not
	14b	Why the trial ended or was stopped	Not
Baseline data	15	A table showing baseline demographic and clinical characteristics for each group	
Numbers analysed	16	For each group, number of participants (denominator) included in each analysis and whether the analysis was by original assigned groups	Not
Outcomes and estimation	17a	For each primary and secondary outcome, results for each group, and the estimated effect size and its precision (such as 95% confidence interval)	Not
	17b	For binary outcomes, presentation of both absolute and relative effect sizes is recommended	Not
Ancillary analyses	18	Results of any other analyses performed, including subgroup analyses and adjusted analyses, distinguishing pre-specified from exploratory	Not
Harms	19	All important harms or unintended effects in each group (for specific guidance see CONSORT for harms)	Not
**Discussion**			
Limitations	20	Trial limitations, addressing sources of potential bias, imprecision, and, if relevant, multiplicity of analyses	No.11
Generalisability	21	Generalisability (external validity, applicability) of the trial findings	No.10
Interpretation	22	Interpretation consistent with results, balancing benefits and harms, and considering other relevant evidence	No.10-11
**Other information**			
Registration	23	Registration number and name of trial registry	No.1
Protocol	24	Where the full trial protocol can be accessed, if available	The article
Funding	25	Sources of funding and other support (such as supply of drugs), role of funders	No.11

*We strongly recommend reading this statement in conjunction with the CONSORT 2010 Explanation and Elaboration for important clarifications on all the items. If relevant, we also recommend reading CONSORT extensions for cluster randomised trials, non-inferiority and equivalence trials, non-pharmacological treatments, herbal interventions, and pragmatic trials. Additional extensions are forthcoming: for those and for up to date references relevant to this checklist, see www.consort-statement.org.

For peer review only - http://bmjopen.bmj.com/site/about/guidelines.xhtml

BMJ Open

Effect of a family-involvement aerobic combined resistance exercise on cancer-related fatigue in patients with Breast Cancer during postoperative chemotherapy:study protocol for a quasi-randomized controlled trial

Journal:	*BMJ Open*
Manuscript ID	bmjopen-2022-064850.R1
Article Type:	Protocol
Date Submitted by the Author:	17-Nov-2022

Complete List of Authors:	Huang, Chuhan; First Affiliated Hospital of Dalian Medical University, Department of Nursing<br>Cai, Yingjie; First Affiliated Hospital of Dalian Medical University, Department of Nursing<br>Guo, Yufei; First Affiliated Hospital of Dalian Medical University, Department of Nursing<br>Jia, Jingjing; First Affiliated Hospital of Dalian Medical University, Department of Nursing<br>Shi, Tieying; First Affiliated Hospital of Dalian Medical University, Department of Nursing

**Primary Subject Heading**:	Nursing
Secondary Subject Heading:	Nursing
Keywords:	Breast tumours < ONCOLOGY, SOCIAL MEDICINE, SPORTS MEDICINE

SCHOLARONE™
Manuscripts

Title of the article: Effect of a family-involvement aerobic combined resistance exercise on cancer-related fatigue in patients with Breast Cancer during postoperative chemotherapy: study protocol for a quasi-randomized controlled trial

Full name, postal address and e-mail of the corresponding author:
Tieying Shi, Department of Nursing, The First Affiliated Hospital of Dalian Medical University, Dalian, China , sty11177@163.com

Tel: +86 0411 83635963

Fax: +86 0411 83622844

Tieying Shi is corresponding authors

Full name, department, institution, city and country of all co-authors:
Chuhan Huang,[1] Yingjie Cai,[1] Yufei Guo,[1] Jingjing Jia,[1] Tieying Shi,[1]
[1]Department of Nursing, The First Affiliated Hospital of Dalian Medical University, Dalian, China

Keywords:
breast cancer; cancer-related fatigue; postoperative chemotherapy; family-involvement; aerobic combined resistance exercise

Word count, excluding title page, abstract, references, figures and tables:
5858

For peer review only

Effect of a family-involvement aerobic combined resistance exercise on cancer-related fatigue in patients with Breast Cancer during postoperative chemotherapy: study protocol for a quasi-randomized controlled trial

Chuhan Huang,[1] Yingjie Cai,[1] Yufei Guo,[1] Jingjing Jia,[1] Tieying Shi,[1]

ABSTRACT

Introduction

Cancer-related fatigue (CRF) is one of the most common and debilitating side effects in patients with breast cancer (BC) throughout postoperative chemotherapy. Family-involvement aerobic combined resistance exercise has been supported as a promising non-pharmacological intervention for the individual symptom relief of cancer-related fatigue, muscle strength, exercise completion, family intimacy and adaptability, and quality of life. However, relevant evidence of using family-involvement aerobic combined resistance exercise for CRF management in patients with BC is lacking report.

Methods and analysis

This study is a quasi-randomized controlled trial involving an 8-week intervention. Seventy patients with BC is recruited from a tertiary medical center in China. The participants from the first oncology department is assigned to the family-involvement aerobic combined resistance exercise group (n=35), while the participants from the second oncology department is assigned to the control group with standard exercise guidance(n=35). The primary outcome is evaluated by the Piper Fatigue Scale-Revised (R-PFS). The secondary outcomes include muscle strength, exercise completion, family intimacy and adaptability, and quality of life, which are evaluated by the stand-up and sit-down chair test, the grip test, the exercise completion rate, the Family Adaptability and Cohesion Scale, Second Edition - Chinese Version (FACESⅡ-CV) and the Functional Assessment of Cancer Therapy - Breast (FACT-B). Analysis of covariance is adopted for comparisons between groups and paired t-test is used for before exercise and after-exercise comparisons within a group.

Key Words breast cancer; cancer-related fatigue; postoperative chemotherapy; family-involvement; aerobic combined resistance exercise

Ethics and dissemination

Ethics approval is obtained from relevant site (PJ-KS-KY-2021-288). The findings of this study is published in a peer-reviewed scientific journal.

Trail registration number

ChiCTR2200055793

Strengths and limitations of this study

For peer review only - http://bmjopen.bmj.com/site/about/guidelines.xhtml

- In this intervention, each participant can choose the type of aerobic exercise they prefer.
- This study will provide exercise instruction videos to increase patients' exercising motivation. An exercise booklet is designed and provided to the participants in both intervention and control groups.
- Given the limited study sites, the sample in this study may only reflect the characteristics of this location .
- Due to the visible nature of the exercise intervention, the blinding of the participants and the investigators cannot be performed in this study which is not a rigorous randomized controlled trial, which might increase the risk of detection bias during the study's implementation.
- The study lacks a long-term follow-up to assess the ongoing effects of family-involvement exercise might be another limitation.

INTRODUCTION

According to the latest data in 2020, the number of new BC patients reached 2.26 million and the number of deaths is 680,000. BC officially replaced lung cancer as the largest cancer worldwide, ranking first in female cancer deaths[1]. In China, there are about 368,000 new cases of BC in 2018, and about 416,000 new cases of BC in 2020[2], showing an increasing trend year by year[3]. And at present, breast cancer patients in China tend to be younger[4]. The number of cancer survivors has increased, and the 5-year survival rate of BC patients has reached 90%, this has been attributed to the progress made in the realm of screening, diagnosis and therapeutic strategies engaged in breast cancer management[5].

As a common adjuvant therapy after BC surgery, chemotherapy can improve the survival rate and prolong the survival time of BC patients[6,7]. However, patients with low immunity during postoperative chemotherapy are prone to various discomforts, which increase their physical and mental burden and reduce their quality of life. Cancer-related fatigue (CRF) is one of the most common symptoms[8]. According to research reports[9], 80% to 90% of cancer patients will experience cancer-related fatigue during treatment and last for a long time. Cancer-related fatigue not only increases the risk of accidental falls in BC patients but also reduces their tolerance to chemotherapy drugs and increases the incidence of adverse reactions such as nausea and vomiting10]. In addition, compared with other symptoms such as pain and depression caused by cancer, cancer-related fatigue has a great negative impact on patients' social barriers and social re-employment[11]. Studies have shown that cancer-related fatigue in patients is positively related to muscle strength[12] and health-related quality of life[13]. Therefore, it is necessary to improve cancer-related fatigue in BC patients to promote their return to society and improve their quality of life.

For peer review only - http://bmjopen.bmj.com/site/about/guidelines.xhtml

Studies have shown that increasing physical activity in BC patients can improve cancer-related fatigue[14]. Physical activity refers to any physical movement that results in energy expenditure caused by skeletal muscle contraction[15], including exercise training such as aerobic exercise, resistance exercise, balance exercise, and flexibility exercise. Adult breast cancer patients aged 18-65 should engage in regular physical activity at the time and intensity recommended by the guidelines[16]. Aerobic exercise and resistance exercise, as the most common exercise training methods in physical activity, have been proved to be safe and effective[17]. Studies have found that aerobic exercise combined with resistance exercise has the best effect in improving cancer-related fatigue[18].

Although the benefits of aerobic combined resistance exercise for cancer-related fatigue have been proven, BC patients do not exercise well. Good external support is one of the important factors for patients to adhere to exercise[19]. As an important part of external support for patients, family members are the main bearers of daily activities and disease care during hospitalization and after discharge[20]. And studies have shown that, compared with unsupervised exercise training, supervised exercise training with the participation of family members can better ensure the continuous and regular development of exercise, thereby maintaining and improving exercise effects[21, 22]. In addition, increasing disease-related communication between family members and patients can effectively increase patients' confidence in treatment, improve the intimacy and adaptability between patients and their families, and thus improve patients' family functions[23]. Therefore, how to play the active support role of family members in the process of exercise training in BC patients, and then enhance the benefits of exercise in improving cancer-related fatigue in patients is one of the urgent problems to be solved.

The current study, therefore, proposes to assess the feasibility and the preliminary effects of using a family-involvement exercise protocol for alleviating the CRF in patients with BC through a quasi-randomized Controlled Trial(Q-RCT).

METHODS AND MATERIALS

Participant Recruitment and Eligibility Criteria

Patients with postoperative chemotherapy of BC in a Grade A tertiary hospital in Dalian are selected for this study.

The inclusion criteria for patients are: (1) pathological diagnosis of BC; (2) with first postoperative chemotherapy for BC; (3) aged 18 to 65; (4) able to cooperate actively; (5) informed consent to participate in this study voluntarily, and (6) with family members during the intervention for 2 months. The exclusion criteria are: (1) with other serious diseases, such as cardiovascular disease, other cancers, etc.; (2) with exercise contraindications, such as asthma, severe anemia, disc herniation or other diseases; (3) with mental illness, previous mental illness or family medical history.

For peer review only

Each patient corresponds to one family member, the inclusion criteria for family members are: (1) aged above 18, with primary school education or above, and good communication skills; (2) spouse, parents, or children of immediate family; (3) the main caregivers determined by the patient and family members who take care of patients above 2 months; (4) voluntary participation in the investigator. The exclusion criteria are: (1) with remuneration; (2) with a previous history of mental illness.

Sample size

According to the previous study[24], cancer-related fatigue score is expected to decrease by 0.6 points. According to the two-sided sample size test formula, a two-sided test with alpha=0.05, and 80% power, this gives a total sample of 30. To account for a 15% attrition rate, the required sample is 70.

Study Design

To avoid contamination among the research subjects, this study adopts a lottery method to randomly divide the hospital's first and second oncology departments into a control group and an intervention group, and select 35 patients in each ward who meet the requirements after screening by the inclusion and exclusion criteria.

The intervention group consist of a sports rehabilitator, a senior clinical nursing specialist, a head nurse, three responsible nurses, and a nursing graduate student. The sports rehabilitator participates in the formulation of the exercise program. The nursing specialist participate in the guidance of the experimental program. The head nurse and three nurses are responsible for quality control, finding problems in the implementation of the program, and making rectifications. While the graduate student is responsible for the intervention of patients and their families, as well as data collection and collation. Group members jointly develope an exercise instruction manual (including exercise forms, exercise methods, exercise precautions, etc.), and record an exercise instruction video.

The researcher explains the purpose and implementation process of the study to the subjects when they are admitted to the hospital for the first chemotherapy (T0). After their agreement to participate, the participants will be required to provide their written informed consent. The participants will be informed that they can withdraw from the study at any moment without any consequences. After obtaining the informed consent of the subjects, the researcher conducts the stand-up and sit-down chair test and the grip test to patients and issues general information questionnaires, R-PFS, FACESⅡ-CV, and FACT-B. The intervention study begins after the patients (and their families) have completed their first assessment.

According to previous relevant studies[25,26], exercise training begins to play a role in “improving cancer-related fatigue” during the 8-week exercise intervention process. Therefore,

For peer review only - http://bmjopen.bmj.com/site/about/guidelines.xhtml

the patients are tested with the stand-up and sit-down chair test and the grip test at the time of the fourth (T1) and eighth (T2) weekends of the intervention. The exercise completion rate of the two groups is evaluated, and the researcher issues general information questionnaires, R-PFS, FACES Ⅱ-CV, and FACT-B to evaluate the effect of exercise intervention. In the process of distributing the questionnaire, if patients could not understand the questionnaire items, the researcher explained it. A Consolidated Standards of Reporting Trials flow chart of the study is presented in figure 1. The schedule of trial enrolment, intervention data collection, and assessments are presented in table 1.

Table1 The schedule of tiral enrolment, interventions and assessments

Study period	Beginning of intervention (week 0)	Interxention period (week 4)	End of intervention (week 8)
Inclusion/exclusion criteria	×		
Informed consent	×		
Demograghic characteristics	×		
Randomisation and allocation	×		
R-PFS	×	×	×
The stand-up and sit-down chair test	×	×	×
The grip test	×	×	×
The exercise completion rate		×	×
FACES Ⅱ-CV	×	×	×
FACT-B	×	×	×

Table1 The schedule of trial enrolment, interventions, and assesments. R-PFS, the Piper Fatigue Scale-Revised; FACES Ⅱ-CV, the Family Adaptability and Cohesion Scale, Second Edition - Chinese Version; FACT-B, the Functional Assessment of Cancer Therapy - Breast

Control group

When patients are admitted to the hospital for the first chemotherapy, the researcher provides exercise guidance for them with “aerobic combined resistance exercise”, and guides patients exercise training through the manual and video, and informs exercise time, form, method, intensity as well as precautions. The patients are followed up monthly to understand their exercise status, and the researcher asks patients why they don’t persist in exercising to help them solve their problems, and to encourage them to persist in exercise training.

Intervention group

Based on the control group, “aerobic combined resistance exercise” with family members is presented in Table 2:

For peer review only - http://bmjopen.bmj.com/site/about/guidelines.xhtml

Guide family members		Evaluate the patients' life and social environment through family members, and explain to family members the importance of family members accompanying the patient to exercise.
		Distribute exercise manuals to family members and play exercise instruction videos.
		Instruct family members to master the exercise time, method, exercise standard posture, exercise precautions, and precautions for the use of elastic bands and dumbbells for BC patients.
Supervise family members	Issue supervision diaries to family members[1].	Remind and supervise family members to fill in the supervision diary through WeChat or phone every month.
		When the patient is readmitted to the hospital, he is required to carry the supervision diary to check the record in the diary.
	Supervise family members via WeChat.	Establish a special WeChat group for family members, send exercise-related health knowledge to the WeChat group every week, and regularly remind family members to urge patients to exercise.
		Encourage family members to ask questions actively in the WeChat group and provide timely feedback on the supervision situation and feelings, to answer relevant questions for family members in the process of guiding and supervising patients.
		Appropriate praise and rewards are given to the family members who first complete the monitoring goals every week, forming a competition mechanism and stimulating interest.
Follow up with family members		Make phone calls or face-to-face follow-up with family member monthly, ask them the questions they encountered during the care of the patients and answer them.
		Ask the patient about the training situation. If patients cannot insist on exercise training, further inquire and analyze the reasons why patients cannot insist, and help the family to solve the problem.
Family members accompany patients		Participate in the whole process of the patients' exercise and provide the patients with full emotional support. Before exercising, choose the way of aerobic exercise together with the patients according to the patient's preference and play music for patients to relax while they exercise.

For peer review only

Family members supervise patients	Give exercise guidance to the patient. Guide the patients in the correct exercise method by playing the video. When the patients make an error or non-standard movement during the exercise, correct it in time.
	Supervise the patient exercising. Take photos or videos while the patients are moving to record the patients' movement. Keep a supervision diary after each patient's exercise and upload exercise photos or videos in the WeChat group to ensure the effect of family supervision.
Family members encourage patients	Encourage and support the patients and enhance their confidence; encourage the patients to tell themselves about the difficulties encountered during exercise, and tell them that they will overcome it together.

[1]The first part of the supervision diary is basic information, including initials of family members, initials of patients, the relationship between family members and patients, height, weight, initials of the doctor in charge, date of chemotherapy, etc. This part is filled out by the researcher and distributed to the family members. The second part includes the exercise date, time, location, exercise duration, frequency and number of groups, the patient's exercise status, and whether he or she participated in the exercise guidance and supervision throughout the exercise.

Table 2 The exercise training intervention with family members

Aerobic combined resistance exercise

The exercise training lasts for 8 weeks, and the patients can select the most comfortable period of the week according to the patients' situation every week. Each training time is scheduled for half an hour after a meal, and avoid training on an empty stomach or a full meal. Each workout includes aerobic and resistance exercises which both are progressive. It is allowed 5 minutes of rest between aerobic and resistance exercise. Aerobic exercise is accomplished by cycling, treadmill, or brisk walking on the ground, depending on the patients' preference: twice a week for 15 to 20 minutes in week1-4; three times a week for 25 to 35 minutes in week 5-8. Resistance exercise used 0.45kg dumbbells or a mineral water bottle filled with 500ml of water and a 1.8cm brown elastic band named TheraBand and provided by the researchers as the main exercise tools, including four resistance exercise movements (hip abduction, seated knee raising, raise before standing, standing lateral raise): twice a week in week 1-4, two sets of each exercise, each set of 8-12 times; three times a week in week 5-8, three sets of each exercise, each set of 8-12 times, with plenty of rest between sets[27-30].

Hip abduction: When preparing, the body is in a standing position, the ankle of one foot is covered with the elastic band, the other foot is on the elastic band, the opposite hand holds the seat, and the supporting foot firmly grasps the ground to keep the trunk stable; at the beginning The buttocks exert force and pull the elastic band to the side until the angle between the practice leg and the supporting foot is about 30 degrees, and pause for about one second, then slowly restore, alternating with the left and right feet.

For peer review only - http://bmjopen.bmj.com/site/about/guidelines.xhtml

Seated knee raising: When preparing, sit in a sitting position with a straight back, hold the seat with both hands, keep the upper torso upright, step on one end of the elastic band with one foot, and put the ankle of one foot on the elastic band to bear the weight; lift the knee at the beginning To the maximum angle of joint activity, and pause for about one second, then slowly lower, alternating left and right.

Raise before standing: Stand with feet parallel, head straight and look straight ahead, hold dumbbells at both sides of the body with both hands, raise arms in front of the body, pause for about one second, and then slowly restore. Inhale during the horizontal lift, exhale when returning to the original position.

Standing lateral raise: Stand with feet parallel, head straight, look straight ahead, hold dumbbells on both sides of the body, raise arms by the side, bend the elbows slightly, focus on the shoulders, and focus on the shoulders. When the arm is raised to a horizontal position, pause for about one second, slowly lower it, and restore the arm to the initial position. Inhale during the horizontal lift, exhale when returning to the original position.

The intensity of aerobic exercise should be moderate, that is, the Borg subjective fatigue scale score should reach 13-14 points (feeling a little hard); the intensity of resistance exercise should be tolerated by the patient. If there is obvious fatigue and muscle pain during or after training, the intensity of physical activity should be appropriately reduced. If patients have severe pain, discomfort and other symptoms occur, they should immediately stop the activity and be admitted to the hospital for re-examination[31,32].

During the exercise, pay attention to observe the patients' reaction and adjust the elastic band or exercise speed and intensity in time; pay attention to the patients' exercise force should not be too large to avoid stretching the wound or the place where the catheter is placed; pay attention to the patient's movement at any time, if the patients are dizzy, discomfort and other adverse events occur, they should be promptly instructed to stop exercising and visit the hospital if necessary.

Outcome measurements

The outcome measurements for this pilot will include two categories, namely, baseline assessments and clinical outcomes.

Demographic and clinical characteristics of the participants

A self-designed demographic and clinical data form is employed to collect the participants' sociodemographic data (eg, age, education background, employment status, marital status, and household income), the participants' medical history (eg, date of diagnosis, the current stage of BC and date and type of treatment) and family members' sociodemographic data at baseline (T0).

Primary outcomes: cancer-related fatigue

The Piper Fatigue Scale-Revised (R-PFS)[33]: The R-PFS is adopted to assess the participants' subjective fatigue. This self-administered questionnaire contains 22 items whose

For peer review only - http://bmjopen.bmj.com/site/about/guidelines.xhtml

scores range from 0 to 10 and includes four domains of subjective fatigue. The R-PFS is available in a simplified Chinese version[34], with high reliability and validity and has high reliability in the BC population.

Secondary outcomes: muscle power, exercise completion, family intimacy and adaptability and QOL

The muscle strength exercise completion, family intimacy and adaptability, and QoL of the patients with BC as the secondary outcomes are measured at T0, T1, and T2 using the stand-up and sit-down chair test, the grip test, the exercise completion rate, the FACESⅡ-CV and the FACT-B.

1. The stand-up and sit-down chair test (number of times standing up from the chair within 30 s) [35]: This test is used to evaluate the leg strength of the subject. The procedure of testing: 1) Put an upright chair (or a folding chair) against the wall (for the sake of safety). 2) The subjects sit in the middle of the chair with the right foot and the left foot apart equal to the shoulders, and one foot may be put slightly front and the other slightly back. Both arms are crossed around the waist and close to the chest. 3) During the test, the subjects should completely stand up and then completely sit down. 4) Record the number of times that the subjects stand up from and sit down in the chair within 30 s. 5) For safety purposes or when necessary, the subject may use her arms for assistance.

2. The grip test[32]: This test is used to evaluate the arm strength of the subject. Use domestic CAMRY (model EH101) electronic grip strength meter, this grip strength meter is in kg/Ib, the maximum range is 90kg/198Ib, and the division value is 0.1kg/0.2Ib. The subjects are in a standing position, with their hands hanging down naturally, their feet are flush with both shoulders, and the hands are not close to the body.

3. Exercise completion rate: This test is used to assess the performance of the two groups of exercise. The exercise completion rate in this study = the number of subjects in the intervention group who completed 18 or more training sessions (over 90% of the total training volume) ÷ the total number of people in the intervention group × 100%.

4. The Family Adaptability and Cohesion Scale, Second Edition - Chinese Version (FACESⅡ-CV)[36]: The FACESⅡ-CV is adopted to assess the subjects' family intimacy and adaptability. A higher score demonstrates higher intimacy and adaptability. The FACESⅡ-CV is available in a simplified Chinese version, with high reliability and validity.

5. The Functional Assessment of Cancer Therapy - Breast (FACT-B)[37]: The FACT-B is adopted to assess the subjcets' QoL. A higher score demonstrates better QoL. The FACT-B is available in a simplified Chinese version, with adequate psychometric properties reported among patients with BC.

Data analysis

For peer review only - http://bmjopen.bmj.com/site/about/guidelines.xhtml

The study results underwent statistical analysis with SPSS software version 25.0. Analysis of covariance is adopted for comparisons between groups and paired t-test is used for before exercise and after-exercise comparisons within a group, whereas $p < 0.05$ represents there is a significant difference between the test results.

Data management

After data collection is complete, all paper data is converted to electronic data. All data is independently recorded by two researchers using Excel. The software automatically checks inconsistent or problematic data based on the inspection results and generates a data problem table. After all, data have been confirmed, reconciled, and stored in an electronic database, participants' identifying information (eg, real name) will not appear in the relevant reports of the trial to protect their privacy. Only researchers directly involved in the analysis of this study have access to the final trial dataset, which contains only coded data.

Data sharing statement

Technical appendix, statistical codes and dataset will be available at any time by contacting the corresponding author.

Patient and public involvement

Patients or the public were not involved in the design, or conduct, or reporting, or dissemination plans of our research.

Ethics and dissemination

Ethical approval is granted by the Ethics Committee of the First Affiliated Hospital of Dalian Medical University (PJ-KS-KY-2021-288) and is retrospectively registered in the Chinese Clinical Trial Registry (registration number: ChiCTR2200055793). Only the participants who signed the informed consent document are included in the study.

DISCUSSION

As one of the most common symptom clusters in patients with BC, the CRF can significantly deteriorate patients' QoL and daily functioning[38]. An increasing number of studies have demonstrated that aerobic combined resistance exercise has beneficial effects on symptom management in patients with cancer[39]; however, due to the time and intensity of exercise and individual differences, the effect of each study varies. Kajal Gokal[40] conducted a home-based walking intervention based on self-management in 25 BC patients receiving chemotherapy. The results showed that self-management exercise intervention can improve patients' fatigue symptoms and improve their physical activity level. However, some patients are difficult to adhere to exercise training, causing the patients to stop exercising. Kyeong[41] conducted a 12-week randomized controlled trial on 356 BC patients. The results showed that aerobic combined resistance exercise can improve patients' fatigue symptoms, muscle strength, and quality of life,

For peer review only - http://bmjopen.bmj.com/site/about/guidelines.xhtml

but because some patients cannot persist in exercise, the advantages of exercise over traditional exercise programs are not obvious. Existing studies have confirmed that family-involvement exercise interventions have many benefits in stroke[42], coronary heart disease[43], lung cancer[44], and other patients, while there are few reports on the effect of family-involvement exercise interventions on BC patients in China. This highlights a great need to explore the effects of family-involvement exercise on the CRF in patients with BC.

The principal strength (and novel aspect) of this study resides in the fact that in the intervention, each participant chooses the type of aerobic exercise they prefer. And the study provides exercise instruction videos to increase patients' exercising motivation. In addition, we aim to provide new evidence on the cost-effectiveness of this kind of intervention (to our knowledge, virtually no evidence has previously been reported in this regard). Moreover, an exercise booklet is designed and provided to the participants in both the intervention and control groups. The information listed in this booklet is comprehensively adapted from relevant national guidelines, professional bodies, and research evidence in published peer-reviewed articles.

This study also has some limitations. Given the limited study sites, the study sample in this study may not offer a completely representative sample of patients with BC. Due to the visible nature of the exercise intervention, the blinding of the participants and the investigators cannot be performed in this study, which might increase the risk of detection bias during the study's implementation. The lack of long-term follow-up to assess the ongoing effects of family-involvement exercise might be another limitation, but this can be considered in the future full-scale trial as one of the main study outcomes. Furthermore, this study is not a rigorous randomized controlled trial, and a future multicentre large-scale main RCT should be conducted in the future to further summarize the research evidence on the effects of family-involvement exercise on the management of patients with CPF.

This study will use a quasi-randomized controlled trial to assess the feasibility and preliminary effects of a family-involvement exercise program for alleviating the CRF in patients with BC under postoperative chemotherapy. The convenience of the family-involvement exercise for the management of the CRF may provide patients with BC, healthcare professionals, and policy-makers with further guidance in CRF management in the long run.

Author affiliations

[1]Department of Nursing, The First Affiliated Hospital of Dalian Medical University, Dalian, China

Contributorship statement Chuhan Huang conceived and designed the study, and Tieying Shi oversaw the research team and research process. Yingjie Cai and Chuhan Huang were the main implementers of the study and drafted the manuscript. Jingjing Jia wrote the ethics review

For peer review only - http://bmjopen.bmj.com/site/about/guidelines.xhtml

confirmation. Yufei Guo participated in the design of the study and assisted in drafting the manuscript. All authors have read and agree to the final version of the manuscript.

Funding This trial was funded by the Dalian Science and Technology Innovation Fund Science and Technology Benefit People Project, and the Award/Grant number is 2022JJ13FG109. The authors have not declared a specific grant for this research from any funding agency in the public, commercial or not-for-profit sectors.

Competing interests None declared.

Patient consent for publication Not required.

Provenance and peer review Not commissioned; externally peer reviewed.

Ethics approval The study has been approved by the Ethics Committee of the First Affiliated Hospital of Dalian Medical University, China(PJ-KS-KY-2021-288).

REFERENCES:

[1]Sung H,Global cancer statistics 2020: GLOBOCAN estimates of incidence and mortality world wide for 36 cancers in 185 countries[J]. A Cancer Journal for Clinicians. 2021, 71(3): 209-249

[2]World Health Organization. Global Cancer Observatory(GCO):Cancer Today[EB/OL] https://gcoiarcfr/today/home, 2021-02-03.

[3]Chen W, Zheng R, Baade PD, et al. Cancer statistics in China, 2015[J]. A Cancer Journal for Clinicians. 2016 ,66(2):115-32.

[4]Jiang Xiaoting, Liu Qiang, Song Erwei. Questions and reflections in the diagnosis and treatment of younger breast cancer in China[J]. National Medical Journal of China, 2022, 102(36): 2823-2827.

[5]Li T, Mello-Thoms C, Brennan PC. Descriptive epidemiology of breast cancer in China: incidence, mortality, survival and prevalence[J]. Breast Cancer Research and Treatment, 2016, 159(3): 395-406.

[6]Breast Cancer Professional Committee of China Anti-Cancer Association. Chinese Anti-Cancer Association Guidelines and Standards for Breast Cancer Diagnosis and Treatment (2021 edition)[J].China Oncology, 2021, 31(10): 954-1040.

[7]Peng Z, Wei J, Lu X, et al.Diagnosis and treatment pattern among rural and urban breast cancer patients in Southwest China from 2005 to 2009[J]. Oncotarget. 2016 , 7(47): 78168-78179.

[8]Sue Kim, Jeehee Han, Min Young Lee, et al. The experience of cancer-related fatigue, exercise and exercise adherence among women breast cancer survivors: Insights from focus group interviews[J]. Journal of Clinical Nursing, 2020, 29(5-6): 758-769.

[9]Fabi A, Bhargava R, Fatigoni S, et al. Cancer-related Fatigue: Esmo Clinical Practice Guidelines for Diagnosis and Treatment[J]. Annals of Oncology, 2020, 31(6): 713-723.

[10]Abrahams HJG, Gielissen MFM, Schmits IC, et al. Risk factors, prevalence, and course of severe fatigue after breast cancer treatment: a meta-analysis involving 12 327 breast cancer survivors[J]. Annals of Oncology, 2016, 27(6): 965-974.

[11]Ekenga CC, Perez M, Margenthaler JA, et al. Early-stage Breast Cancer and Employment Participation after 2 Years of Follow-up: A Comparison with Age-matched Controls[J]. Cancer, 2018, 124(9): 2026-2035.

For peer review only - http://bmjopen.bmj.com/site/about/guidelines.xhtml

[12]Mustian KM, Alfano CM, Heckler CE, et al. Comparison of pharmaceutical, psychological, and exercise treatments for cancer-related fatigue: a meta-analysis[J]. JAMA Oncology, 2017, 3(7):961-968.
[13]Ehlers Diane K, DuBois Kelli, Salerno Elizabeth A. The effects of exercise on cancer-related fatigue in breast cancer patients during primary treatment: a meta-analysis and systematic review[J]. Expert Review of Anticancer Therapy, 2020, 20(10): 865-877.
[14]Lahart Ian M, Metsios George S, Nevill Alan M, et al. Physical activity for women with breast cancer after adjuvant therapy[J]. The Cochrane database of systematic reviews, 2018, 1(1): CD011292.
[15]Caspersen CJ, Powell KE, Christenson GM. Physical activity, exercise, and physical fitness: definitions and distinctions for health-related research[J]. Public Health Reports, 1985, 100(2): 126-131.
[16]Breast Health Group(BEST: Breast Education Screening Diagnosis and Treatment Group) of the Branch of Women Health of Chinese Preventive Medicine Association. Guidelines on life-style modification for Chinese breast cancer surviors[J]. Chinese Journal of Surgery, 2017, 55(2): 81-85.
[17]Joel T Fuller, Michael C Hartland, Luke T Maloney, et al. Therapeutic effects of aerobic and resistance exercises for cancer survivors: a systematic review of meta-analyses of clinical trials[J]. British Journal of Sports Medicine,2018, 52(20): 1311.
[18]ZHOU Shuai, JIANG Jinfang, ZHANG Ling, et al. Different Exercise Therapies in Patients with Cancer-related Fatigue: A Network Meta-Analysis[J].Nursing Journal of Chinese People's Liberation Army, 2021, 38(08): 65-68+88.
[19]DENG Yangqing, PAN Cen, WANG Qinglan,et al.Qualitative Research on Exercise Experience of Patients with Breast Cancer: A Meta-synthesis[J].Journal of Nursing(China), 2021, 28(13): 34-39.
[20]Liu Min, Li Guohong, Xue Xiaoming, et al. Effect of family-participated continuation nursing model on the quality of life of discharged patients with colorectal cancer[J].Chinese Journal of Gerontology, 2020, 40(23): 5080-5085.
[21]Wanderson Divino Nilo dos Santos, Amilton Vieira, Claudio Andre Barbosa de Lira, et al. Once a Week Resistance Training Improves Muscular Strength in Breast Cancer Survivors: A Randomized Controlled Trial[J]. Integrative Cancer Therapies,2019,18, 1534735419879748..
[22]Witlox L, Velthuis MJ, Boer JH, et al. Attendance and compliance with an exercise program during localized breast cancer treatment in a randomized controlled trial: The PACT study[J]. PLoS One,2019, 14(5): e0215517.
[23]YUN Qingping, JI Ying, JIANG Xuewen, et al.Path analysis of health behavior improvement in chronic disease patients based on family function theory[J].Chinese Journal of Disease Control & Prevention, 2019, 23(07): 861-865.
[24]Zhu Hongyu, Zhang Yunfei, Ke Xi, et al. The effect of graded walking exercise on cancer-related fatigue in patients with gastric cancer during chemo therapy[J].Chinese Journal of Nursing, 2021, 56(05): 652-658.
[25]Jiang Yongqin, Yan Ling, Liu Chunyan, et al. Effects of exercise prescription on cancer-related fatigue in breast cancer patients[J]. Chinese Journal of Nursing, 2008(10): 906-909.
[26]Yan Ling. Effects of exercise prescription on cancer-related fatigue in breast cancer patients[D].Tianjin Medical University, 2008.
[27]Anne Marthe Schreuder, Anne M. Eskes, Rosanna G.M. van Langen, et al. Active involvement of family members in postoperative care after esophageal or pancreatic resection: A

For peer review only

feasibility study[J]. Surgery, 2019, 166(5): 769-777.
[28]Huang HP, Wen FH, Yang TY, et al. The effect of a 12-week home-based walking program on reducing fatigue in women with breast cancer undergoing chemotherapy: A randomized controlled study[J]. International Journal of Nursing Studies, 2019, 11(9): 103376.
[29]Li He, Wang Yixiao, Shi Tieying,et al. Effect of Progressive Resistance Training on Upper Limb Function in Breast Cancer Patients[J].Chinese Journal of Rehabilitation Theory and Practice, 2015, 21(11): 1356-1359.
[30]Qin Bo. Effects of elastic band resistance training on lower limb muscle strength and physical activity in the elderly[D].Shanghai Normal University, 2021.
[31]Dong Xin, Mo Yihan, WANG Xiuhua. Effects of resistance exercise on muscle mass, muscle strength and physical performance in the elderly at risk of sarcopenia[J].Chinese Nursing Management, 2021, 21(08): 1190-1195.
[32]Li He. Postoperative progressive resistance training on the rehabilitation of breast cancer patients[D]. Dalian Medical University, 2015.
[33]So WKW, Dodgson J, Tai JWM. Fatigue and quality of life among Chinese patients with hematologic malignancy after bone marrow transplantation[J]. Cancer Nursing, 2003, 26(3):211-219.
[34]Li L, Zhu LH, Cao MQ. Progress in the diagnosis and evaluation scale of cancer-related fatigue[J]. Xinjiang Medcine Journal, 2016, 46(11): 1458-1461,1465.
[35]Xiaosheng Dong, Xiangren Yi, Dezong Gao, et al. The effects of the combined exercise intervention based on internet and social media software (CEIBISMS) on quality of life, muscle strength and cardiorespiratory capacity in Chinese postoperative breast cancer patients:a randomized controlled trial[J]. BioMed Central,2019,17(1): 109.
[36]Fei Lipeng, Shen Qijie, Zheng Yanping, et al. Preliminary evaluation of “Family Intimacy and Adaptability Scale” and “Family Environment Scale”[J].Chinese Mental Health Journal, 1991(05): 198-202+238.
[37]Wan Chonghua, Tang Xueliang. Introduction to the Chinese version of FACT-B, a measure of quality of life for breast cancer patients[J]. China Cancer, 2002, 11(6):318-320.
[38]Ruiz-Casado A, Álvarez-Bustos A, de Pedro CG, et al. Cancer-related Fatigue in Breast Cancer Survivors: A Review[J]. Clinical Breast Cancer. 2021, 21(1): 10-25.
[39]BOLAM K A, MIJWEL S, RUNDQVIST H, et al. Two-year follow-up of the OptiTrain randomised controlled exercise trial[J]. Breast Cancer Research and Treatment, 2019, 175(3): 637-648.
[40]Gokal Kajal, Wallis Deborah, Ahmed Samreen, et al. Effects of a self-managed home-based walking intervention on psychosocial health outcomes for breast cancer patients receiving chemotherapy: a randomised controlled trial[J]. Supportive care in cancer, 2016, 24(3): 1139-66..
[41]Kyeong Eun Uhm, Ji Sung Yoo, Seung Hyun Chung, et al. Effects of exercise intervention in breast cancer patients: is mobile health (mHealth) with pedometer more effective than conventional program using brochure[J]. Breast Cancer Research and Treatment,2017,161(3):443-452.
[42]Galvin R, Cusack T, Stokes E. A randomised controlled trial evaluating family mediated exercise (FAME) therapy following stroke[J]. BMC Neurology. 2008, 20(8): 22.
[43]Ma Lili, Sun Suping, YAN Xueqing, et al. Effects of cardiac rehabilitation with the participation of spouses on the patients undergoing PCI and their spouses[J].Chinese Nursing Management, 2020, 20(03): 393-398.
[44]Kelly J. Lafaro, Dan J. Raz, Jae Y. Kim, et al. Pilot study of a telehealth perioperative

For peer review only - http://bmjopen.bmj.com/site/about/guidelines.xhtml

physical activity intervention for older adults with cancer and their caregivers[J]. Supportive Care in Cancer,2020,28(8): 3867-3876.

Figure 1 A Consolidated Standards of Reporting Trials flow chart of the study

For peer review only

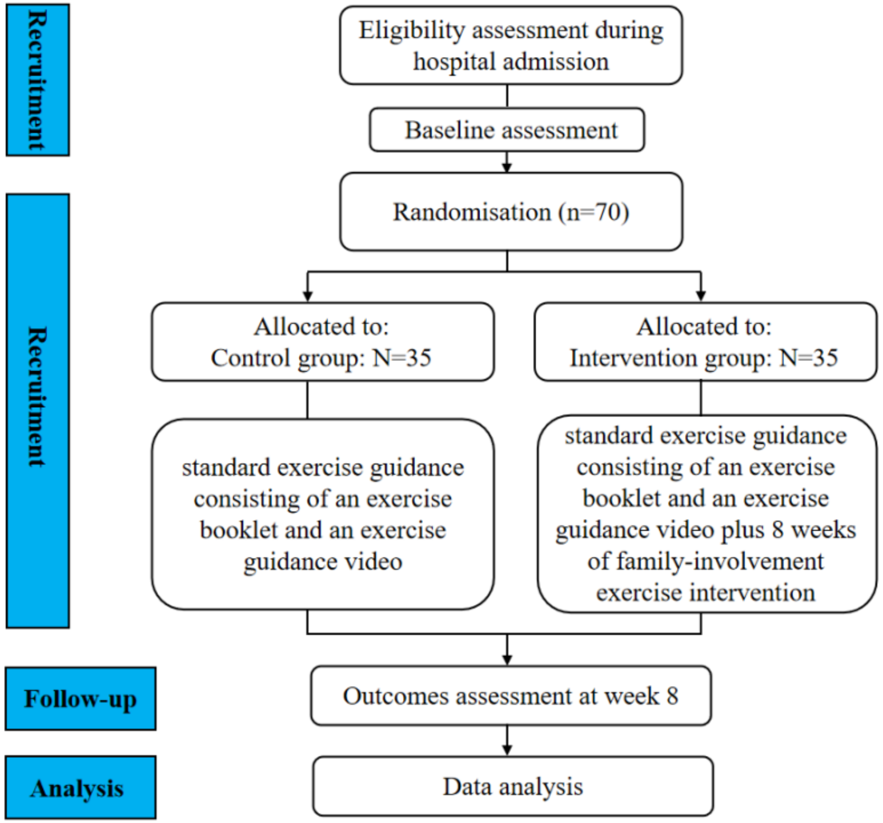
Recruitment
Eligibility assessment during hospital admission
Baseline assessment
Recruitment
Randomisation (n=70)
Allocated to: Control group: N=35
Allocated to: Intervention group: N=35
standard exercise guidance consisting of an exercise booklet and an exercise guidance video
standard exercise guidance consisting of an exercise booklet and an exercise guidance video plus 8 weeks of family-involvement exercise intervention
Follow-up
Outcomes assessment at week 8
Analysis
Data analysis

SPIRIT
STANDARD PROTOCOL ITEMS: RECOMMENDATIONS FOR INTERVENTIONAL TRIALS

SPIRIT 2013 Checklist: Recommended items to address in a clinical trial protocol and related documents*

Section/item	Item No	Description	Reported on page No
**Administrative information**			
Title	1	Descriptive title identifying the study design, population, interventions, and, if applicable, trial acronym	No.1 No.2
Trial registration	2a	Trial identifier and registry name. If not yet registered, name of intended registry	No.2
	2b	All items from the World Health Organization Trial Registration Data Set	
Protocol version	3	Date and version identifier	
Funding	4	Sources and types of financial, material, and other support	No.13
Roles and responsibilities	5a	Names, affiliations, and roles of protocol contributors	No.13
	5b	Name and contact information for the trial sponsor	No.13
	5c	Role of study sponsor and funders, if any, in study design; collection, management, analysis, and interpretation of data; writing of the report; and the decision to submit the report for publication, including whether they will have ultimate authority over any of these activities	No.13
	5d	Composition, roles, and responsibilities of the coordinating centre, steering committee, endpoint adjudication committee, data management team, and other individuals or groups overseeing the trial, if applicable (see Item 21a for data monitoring committee)	No.13
**Introduction**			
Background and rationale	6a	Description of research question and justification for undertaking the trial, including summary of relevant studies (published and unpublished) examining benefits and harms for each intervention	No.3-4
	6b	Explanation for choice of comparators	No.3-4
Objectives	7	Specific objectives or hypotheses	No.4

For peer review only - http://bmjopen.bmj.com/site/about/guidelines.xhtml

Trial design	8	Description of trial design including type of trial (eg, parallel group, crossover, factorial, single group), allocation ratio, and framework (eg, superiority, equivalence, noninferiority, exploratory)	No.5-6
**Methods: Participants, interventions, and outcomes**			
Study setting	9	Description of study settings (eg, community clinic, academic hospital) and list of countries where data will be collected. Reference to where list of study sites can be obtained	No.4
Eligibility criteria	10	Inclusion and exclusion criteria for participants. If applicable, eligibility criteria for study centres and individuals who will perform the interventions (eg, surgeons, psychotherapists)	No.4-5
Interventions	11a	Interventions for each group with sufficient detail to allow replication, including how and when they will be administered	No.6-9
	11b	Criteria for discontinuing or modifying allocated interventions for a given trial participant (eg, drug dose change in response to harms, participant request, or improving/worsening disease)	
	11c	Strategies to improve adherence to intervention protocols, and any procedures for monitoring adherence (eg, drug tablet return, laboratory tests)	
	11d	Relevant concomitant care and interventions that are permitted or prohibited during the trial	
Outcomes	12	Primary, secondary, and other outcomes, including the specific measurement variable (eg, systolic blood pressure), analysis metric (eg, change from baseline, final value, time to event), method of aggregation (eg, median, proportion), and time point for each outcome. Explanation of the clinical relevance of chosen efficacy and harm outcomes is strongly recommended	No.9-11
Participant timeline	13	Time schedule of enrolment, interventions (including any run-ins and washouts), assessments, and visits for participants. A schematic diagram is highly recommended (see Figure)	Table 1
Sample size	14	Estimated number of participants needed to achieve study objectives and how it was determined, including clinical and statistical assumptions supporting any sample size calculations	No.5
Recruitment	15	Strategies for achieving adequate participant enrolment to reach target sample size	No.4-5
**Methods: Assignment of interventions (for controlled trials)**			
Allocation:			

Sequence generation	16a	Method of generating the allocation sequence (eg, computer-generated random numbers), and list of any factors for stratification. To reduce predictability of a random sequence, details of any planned restriction (eg, blocking) should be provided in a separate document that is unavailable to those who enrol participants or assign interventions	
Allocation concealment mechanism	16b	Mechanism of implementing the allocation sequence (eg, central telephone; sequentially numbered, opaque, sealed envelopes), describing any steps to conceal the sequence until interventions are assigned	
Implementation	16c	Who will generate the allocation sequence, who will enrol participants, and who will assign participants to interventions	No.5
Blinding (masking)	17a	Who will be blinded after assignment to interventions (eg, trial participants, care providers, outcome assessors, data analysts), and how	
	17b	If blinded, circumstances under which unblinding is permissible, and procedure for revealing a participant's allocated intervention during the trial	
**Methods: Data collection, management, and analysis**			
Data collection methods	18a	Plans for assessment and collection of outcome, baseline, and other trial data, including any related processes to promote data quality (eg, duplicate measurements, training of assessors) and a description of study instruments (eg, questionnaires, laboratory tests) along with their reliability and validity, if known. Reference to where data collection forms can be found, if not in the protocol	No.5<br>No.9-11
	18b	Plans to promote participant retention and complete follow-up, including list of any outcome data to be collected for participants who discontinue or deviate from intervention protocols	No.6
Data management	19	Plans for data entry, coding, security, and storage, including any related processes to promote data quality (eg, double data entry; range checks for data values). Reference to where details of data management procedures can be found, if not in the protocol	No.11
Statistical methods	20a	Statistical methods for analysing primary and secondary outcomes. Reference to where other details of the statistical analysis plan can be found, if not in the protocol	No.11
	20b	Methods for any additional analyses (eg, subgroup and adjusted analyses)	
	20c	Definition of analysis population relating to protocol non-adherence (eg, as randomised analysis), and any statistical methods to handle missing data (eg, multiple imputation)	

For peer review only - http://bmjopen.bmj.com/site/about/guidelines.xhtml

Methods: Monitoring

Data monitoring	21a	Composition of data monitoring committee (DMC); summary of its role and reporting structure; statement of whether it is independent from the sponsor and competing interests; and reference to where further details about its charter can be found, if not in the protocol. Alternatively, an explanation of why a DMC is not needed	
	21b	Description of any interim analyses and stopping guidelines, including who will have access to these interim results and make the final decision to terminate the trial	
Harms	22	Plans for collecting, assessing, reporting, and managing solicited and spontaneously reported adverse events and other unintended effects of trial interventions or trial conduct	
Auditing	23	Frequency and procedures for auditing trial conduct, if any, and whether the process will be independent from investigators and the sponsor	

Ethics and dissemination

Research ethics approval	24	Plans for seeking research ethics committee/institutional review board (REC/IRB) approval	No.11
Protocol amendments	25	Plans for communicating important protocol modifications (eg, changes to eligibility criteria, outcomes, analyses) to relevant parties (eg, investigators, REC/IRBs, trial participants, trial registries, journals, regulators)	
Consent or assent	26a	Who will obtain informed consent or assent from potential trial participants or authorised surrogates, and how (see Item 32)	No.5
	26b	Additional consent provisions for collection and use of participant data and biological specimens in ancillary studies, if applicable	
Confidentiality	27	How personal information about potential and enrolled participants will be collected, shared, and maintained in order to protect confidentiality before, during, and after the trial	No.13
Declaration of interests	28	Financial and other competing interests for principal investigators for the overall trial and each study site	No.13
Access to data	29	Statement of who will have access to the final trial dataset, and disclosure of contractual agreements that limit such access for investigators	No.13
Ancillary and post-trial care	30	Provisions, if any, for ancillary and post-trial care, and for compensation to those who suffer harm from trial participation	

For peer review only

Dissemination policy	31a	Plans for investigators and sponsor to communicate trial results to participants, healthcare professionals, the public, and other relevant groups (eg, via publication, reporting in results databases, or other data sharing arrangements), including any publication restrictions	No.11
	31b	Authorship eligibility guidelines and any intended use of professional writers	
	31c	Plans, if any, for granting public access to the full protocol, participant-level dataset, and statistical code	No.11
**Appendices**			
Informed consent materials	32	Model consent form and other related documentation given to participants and authorised surrogates	
Biological specimens	33	Plans for collection, laboratory evaluation, and storage of biological specimens for genetic or molecular analysis in the current trial and for future use in ancillary studies, if applicable	

*It is strongly recommended that this checklist be read in conjunction with the SPIRIT 2013 Explanation & Elaboration for important clarification on the items. Amendments to the protocol should be tracked and dated. The SPIRIT checklist is copyrighted by the SPIRIT Group under the Creative Commons "Attribution-NonCommercial-NoDerivs 3.0 Unported" license.

For peer review only

BMJ Open

Effect of a family-involvement combined aerobic and resistance exercise protocol on cancer-related fatigue in breast cancer patients during postoperative chemotherapy: study protocol for a quasi-randomized controlled trial

Journal:	*BMJ Open*
Manuscript ID	bmjopen-2022-064850.R2
Article Type:	Protocol
Date Submitted by the Author:	24-Feb-2023

Complete List of Authors:	Huang, Chuhan; First Affiliated Hospital of Dalian Medical University, Department of Nursing<br>Cai, Yingjie; First Affiliated Hospital of Dalian Medical University, Department of Nursing<br>Guo, Yufei; First Affiliated Hospital of Dalian Medical University, Department of Nursing<br>Jia, Jingjing; First Affiliated Hospital of Dalian Medical University, Department of Nursing<br>Shi, Tieying; First Affiliated Hospital of Dalian Medical University, Department of Nursing

**Primary Subject Heading**:	Nursing
Secondary Subject Heading:	Nursing
Keywords:	Breast tumours < ONCOLOGY, SOCIAL MEDICINE, SPORTS MEDICINE

SCHOLARONE™
Manuscripts

Title of the article: Effect of a family-involvement combined aerobic and resistance exercise protocol on cancer-related fatigue in breast cancer patients during postoperative chemotherapy: study protocol for a quasi-randomized controlled trial

Full name, postal address and e-mail of the corresponding author:

Tieying Shi, Department of Nursing, The First Affiliated Hospital of Dalian Medical University, Dalian, China , sty11177@163.com

Tel: +86 0411 83635963

Fax: +86 0411 83622844

Tieying Shi is corresponding authors

Full name, department, institution, city and country of all co-authors:

Chuhan Huang,[1] Yingjie Cai,[1] Yufei Guo,[1] Jingjing Jia,[1] Tieying Shi,[1]

[1]Department of Nursing, The First Affiliated Hospital of Dalian Medical University, Dalian, China

Keywords:

breast cancer; cancer-related fatigue; postoperative chemotherapy; family-involvement; combined aerobic and resistance exercise

Word count, excluding title page, abstract, references, figures and tables:

3785

For peer review only

For peer review only - http://bmjopen.bmj.com/site/about/guidelines.xhtml

Effect of a family-involvement combined aerobic and resistance exercise protocol on cancer-related fatigue in breast cancer patients during postoperative chemotherapy: study protocol for a quasi-randomized controlled trial

Chuhan Huang,[1] Yingjie Cai,[1] Yufei Guo,[1] Jingjing Jia,[1] Tieying Shi,[1]

ABSTRACT

Introduction: Cancer-related fatigue (CRF) is one of the most common and debilitating side effects experienced by breast cancer (BC) patients during postoperative chemotherapy. Family-involvement combined aerobic and resistance exercise has been introduced as a promising non-pharmacological intervention for CRF symptom relief and improving patients' muscle strength, exercise completion, family intimacy and adaptability, and quality of life. However, evidence for the practice of home participation in combined aerobic and resistance exercise for the management of CRF in BC patients is lacking.

Methods and analysis: We present a protocol for a quasi-randomized controlled trial involving an 8-week intervention. Seventy BC patients will be recruited from a tertiary care center in China. Participants from the first oncology department will be assigned to the family-involvement combined aerobic and resistance exercise group (n=28), while participants from the second oncology department will be assigned to the control group that will receive standard exercise guidance (n=28). The primary outcome will be the Piper Fatigue Scale-Revised (R-PFS) score. The secondary outcomes will include muscle strength, exercise completion, family intimacy and adaptability, and quality of life, which will be evaluated by the stand-up and sit-down chair test, grip test, exercise completion rate, Family Adaptability and Cohesion Scale, Second Edition - Chinese Version (FACESⅡ-CV), and Functional Assessment of Cancer Therapy - Breast (FACT-B) scale. Analysis of covariance will be applied for comparisons between groups, and paired t-tests will be used for comparison of data before and after exercise within a group.

Key Words: breast cancer; cancer-related fatigue; postoperative chemotherapy; family-involvement; combined aerobic and resistance exercise

Ethics and dissemination: This study has been approved by the Ethics Committee of the First Affiliated Hospital of Dalian Medical University (PJ-KS-KY-2021-288). The results of this study will be published via peer-reviewed publications and presentations at conferences.

Trail registration number: ChiCTR2200055793

Strengths and limitations of this study

For peer review only - http://bmjopen.bmj.com/site/about/guidelines.xhtml

- This will be the first clinical study to explore the preliminary effects of a family-involvement combined aerobic and resistance exercise protocol on cancer-related fatigue in breast cancer patients during postoperative chemotherapy.
- The family members of the intervention group will record activities in a supervision diary and check in a WeChat group throughout the intervention to illustrate the role of the family members in the intervention process.
- The main limitation of this study will be the small sample size.
- The findings of this study may only reflect the characteristics of patients in this location given the limited study sites.
- Another potential limitation is that study protocol lacks a long-term follow-up to assess the ongoing effects of family-involvement exercise.

INTRODUCTION

According to the latest data published for 2020, the number of newly diagnosed cases of breast cancer (BC) in that year reached 2.26 million worldwide and the number of deaths due to BC was 680,000. BC has officially replaced lung cancer as the most common cancer worldwide and ranks first among females as the cause of cancer-related deaths[1]. In China, approximately 368,000 new BC cases were diagnosed in 2018, and approximately 416,000 new cases of BC were diagnosed in 2020[2], showing an increasing trend year by year[3]. At present, BC patients in China tend to be younger than those in other populations[4]. Overall, the number of BC survivors has increased, as the 5-year survival rate of BC patients has reached 90%. This has been attributed to the progress made in screening, diagnosis and therapeutic strategies applied in BC management[5].

As a common adjuvant therapy after BC surgery, chemotherapy can improve the survival rate and prolong the survival time of BC patients[6,7]. However, patients with low immunity during postoperative chemotherapy are prone to various discomforts, which increase their physical and mental burden and reduce their quality of life. Cancer-related fatigue (CRF) is one of the most common symptoms experienced by BC patients during postoperative chemotherapy[8]. According to previous research[9], 80%–90% of cancer patients will experience CRF during treatment, and this condition will persist for a long time. Research has also shown that CRF not only increases the risk of accidental falls in BC patients but also reduces their tolerance to chemotherapy drugs and increases the incidence of adverse reactions such as nausea and vomiting[10]. In addition, compared with other symptoms such as pain and depression caused by cancer, CRF has a major negative impact on patients' social interactions and re-employment[11]. Studies have shown that CRF in patients is significantly related to muscle strength[12] and health-related quality of life[13]. Therefore, it is necessary to improve CRF in BC patients to promote their return to society and improve their quality of life.

For peer review only - http://bmjopen.bmj.com/site/about/guidelines.xhtml

Increased physical activity in BC patients has been shown to improve CRF[14]. Physical activity refers to any physical movement that results in energy expenditure caused by skeletal muscle contraction[15], including exercise training such as aerobic exercise, resistance exercise, balance exercise, and flexibility exercise. Adult BC patients aged 18–65 years are recommended to engage in regular physical activity at the intensity and for the duration specified by current guidelines[16]. Aerobic exercise and resistance exercise, as the most common exercise training methods in physical activity, have been proven to be safe and effective[17]. Studies have found that aerobic exercise combined with resistance exercise has the best effect on improving CRF[18].

Although the benefits of combined aerobic and resistance exercise for CRF have been proven, many BC patients do not exercise well. Good external support is one of the important factors for patients' adherence to exercise[19]. As an important part of external support for patients, family members are the main facilitators of daily activities and disease care during hospitalization and after discharge[20]. Studies have shown that, compared with unsupervised exercise training, supervised exercise training with the participation of family members can better ensure the continuous and regular development of exercise, thereby maintaining and improving exercise effects[21, 22]. In addition, increasing disease-related communication between family members and patients can effectively increase patients' confidence in treatment, improve the intimacy and adaptability between patients and their family members, and thereby, improve patients' family functioning[23]. Therefore, there is an urgent need for a method to promote the active support role of family members in the process of exercise training for BC patients, which will then enhance the benefits of exercise in improving CRF in these patients.

The current study, therefore, proposes to assess the preliminary effects of a family-involvement exercise protocol on alleviating CRF in BC patients through a quasi-randomized controlled trial (Q-RCT).

METHODS AND MATERIALS

Participant Recruitment and Eligibility Criteria

Patients receiving postoperative chemotherapy for BC in a Grade A tertiary hospital in Dalian will be selected for this study.

The inclusion criteria will be: (1) pathological diagnosis of BC; (2) receiving first course of postoperative chemotherapy for BC; (3) age 18–65 years; (4) able to cooperate actively; (5) voluntary provision of informed consent to participate in this study; and (6) residing with family members during the intervention for 2 months. The exclusion criteria will be: (1) any other serious disease, such as cardiovascular disease, other type of cancer, etc.; (2) exercise contraindications, such as asthma, severe anemia, disc herniation or other diseases; and (3) mental illness, previous mental illness, or family history of mental illness.

For peer review only - http://bmjopen.bmj.com/site/about/guidelines.xhtml

One family member will be included for each BC patients, and the inclusion criteria for family members will be: (1) age >18 years, with primary school education or above, and good communication skills; (2) spouse, parent, or child of the BC patient (immediate family member); (3) main caregiver as determined by the BC patient and family members for at least 2 months; and (4) voluntary participation in the study. The exclusion criteria will be: (1) payment of remuneration for care of the BC patient; and (2) a previous history of mental illness.

Sample size

According to a previous study[24], the score for CRF is expected to decrease by 2.29 points. According to the two-sided sample size test formula, a two-sided test with alpha=0.05 and 90% power will require a total sample of 23 patients in each of the two groups. To account for a 20% attrition rate, the required sample size is 56.

Study Design

To avoid contamination among the research participants, this study will adopt a lottery method to randomly divide the hospital's first and second oncology departments into a control group and intervention group and then select 28 patients from each ward who meet the requirements after screening by the inclusion and exclusion criteria.

The intervention team will consist of a sports rehabilitator, a senior clinical nursing specialist, a head nurse, three responsible nurses, and a nursing graduate student. The sports rehabilitator will participate in the formulation of the exercise program. The nursing specialist will participate in the guidance of the experimental program. The head nurse and three nurses will be responsible for quality control, identifying problems in the implementation of the program, and making rectifications. The graduate student will be responsible for explaining the intervention to the BC patients and their families as well as data collection and collation. Group members will jointly develop an exercise instruction manual (including exercise form, methods, precautions, etc.) and record an instructional video for the exercise protocol.

The researcher will explain the purpose and implementation process of the study to the BC patients when they are admitted to the hospital for their first chemotherapy treatment (T0). After their agreement to participate, participants will be asked to provide written informed consent. The participants will be informed that they can withdraw from the study at any time without any consequences. After obtaining informed consent from the patients, the researcher will conduct the stand-up and sit-down chair test and grip test with patients and issue general information questionnaires along with the R-PFS, FACESⅡ-CV, and FACT-B. The intervention study will begin after the patients (and their family members) have completed their first assessment.

According to previous relevant studies[25,26], exercise training begins to play a role in improving CRF during the 8-week exercise intervention process. Therefore, the patients will be

For peer review only - http://bmjopen.bmj.com/site/about/guidelines.xhtml

tested with the stand-up and sit-down chair test and the grip test at the time of the fourth (T1) and eighth (T2) weekends of the intervention. The exercise completion rates in the two groups will be evaluated at these time points, and the researcher will again issue the general information questionnaires, R-PFS, FACESⅡ-CV, and FACT-B to evaluate the effects of the exercise intervention. In the process of distributing the questionnaires, if patients are unable to understand the questionnaire items, the researcher will explain the items. A Consolidated Standards of Reporting Trials flow chart for the study is presented in Figure 1. The schedule for trial enrolment, intervention, and assessment is presented in Table 1.

Table 1 Schedule of trial enrolment, intervention and assessment

Study period	Beginning of intervention (week 0)	Intervention period (week 4)	End of intervention (week 8)
**Inclusion/exclusion criteria**	×		
**Informed consent**	×		
**Demographic characteristics**	×		
**Randomization and allocation**	×		
**R-PFS**	×	×	×
**Stand-up and sit-down chair test**	×	×	×
**Grip test**	×	×	×
**Exercise completion rate**		×	×
**FACESⅡ-CV**	×	×	×
**FACT-B**	×	×	×

Abbreviations: R-PFS, Piper Fatigue Scale-Revised; FACESⅡ-CV, Family Adaptability and Cohesion Scale, Second Edition - Chinese Version; FACT-B, Functional Assessment of Cancer Therapy – Breast

Control group

When patients are admitted to the hospital for their first chemotherapy treatment, the researcher will provide exercise guidance for "combined aerobic and resistance exercise" and guide patients through the manual and video for exercise training, informing them of the recommended exercise time, form, method, intensity and precautions. The patients will be followed up monthly to learn their exercise status, and if patients have not persisted in following the recommendations, the researcher will ask patients why in order to help solve their problems and to encourage them to persist in exercise training.

For peer review only - http://bmjopen.bmj.com/site/about/guidelines.xhtml

Intervention group

The family-involvement combined aerobic and resistance protocol to be applied in the intervention group is described in detail in Table 2.

Table 2 Exercise training intervention with family members

Guidance for family members	Evaluate the patients' life and social environment through family members and explain to family members the importance of family members accompanying the patient during exercise.	
	Distribute exercise manuals to family members and play exercise instructional videos.	
	Instruct family members to master the exercise time, method, standard posture and precautions, as well as precautions for the use of elastic bands and dumbbells for BC patients.	
Supervision of family members	Issue supervision diaries to family members[1]	Instruct and remind family members regarding the completion of the supervision diary through WeChat or phone every month.
		Upon readmission to the hospital, the patient will be required to bring the supervision diary in order for the record in the diary to be checked.
	Supervision of family members via WeChat	Establish a special WeChat group for family members, send exercise-related health knowledge to the WeChat group every week and regularly remind family members to urge patients to exercise.
		Encourage family members to ask questions actively in the WeChat group and provide timely feedback on the supervision situation and their feelings, answering relevant questions from family members in the process of guiding and supervising patients.
		Provide appropriate praise and rewards to the family members who complete the monitoring goals every week first, forming a competition mechanism to stimulate interest.
Follow-up with family members	Make phone calls or complete face-to-face follow-up with family member monthly, asking them about questions they encountered during the care of the patients and answering those questions.	
	Ask the patient about the training situation. If patients' exercise completion are poor, further inquire about why and analyze the reasons, helping the family to solve the problem.	
Instructions to family members		

For peer review only - http://bmjopen.bmj.com/site/about/guidelines.xhtml

Family members will accompany patients	Participate in the whole process of the patient's exercise protocol and provide the patient with full emotional support. Before exercising, choose the method of aerobic exercise together with the patient according to the patient's preference and play music to help the patient relax while they exercise.
Family members will supervise patients	Give exercise guidance to the patient, informing the patient of the correct exercise method by playing the provided video. When the patient makes an error or non-standard movement during the exercise, provide timely correction.
	Supervise the patient exercising. Take photos or videos while the patient is moving to record the patient's movement. Keep a supervision diary after each exercise session and upload exercise photos or videos in the WeChat group to ensure the effect of family supervision.
Family members will encourage patients	Encourage and support the patient to enhance their confidence; encourage the patient to describe difficulties encountered during exercise, and ensure them that the patient and family member will overcome the difficulty together.

[1]The first part of the supervision diary includes basic information, including initials of family members, initials of patients, the relationship between family members and patients, patients' height and weight, initials of the doctor in charge, date of chemotherapy, etc. This part is filled out by the researcher before the diary is distributed to the family members. The second part includes the exercise date, time, location, exercise duration, frequency and number of groups, the patient's exercise status, and whether the family member participated in the exercise guidance and supervision throughout the exercise.

Combined aerobic and resistance exercise

The designed exercise training intervention will last for 8 weeks, and each patient can select the most comfortable period of the week according to his or her own situation every week. Each training time is scheduled for half an hour after a meal, and participants are instructed to avoid training with an empty or full stomach. Each workout includes aerobic and resistance exercises that are both progressive. A rest period of 5 minutes is allowed between aerobic and resistance exercise. Aerobic exercise is accomplished by cycling, walking on a treadmill, or brisk walking on the ground, depending on the patient's preference, according to the following schedule: twice per week for 15–20 minutes in weeks 1–4 and three times per week for 25–35 minutes in weeks 5–8. For resistance exercise, the patients will use 0.45-kg dumbbells or a mineral water bottle filled with 500 ml of water and a 18-cm brown elastic band with the brand name TheraBand. These items will be provided by the researchers as the main exercise tools for four resistance exercise movements: hip abduction, seated knee raising, raise before standing, and standing lateral raise. These exercises are to be completed twice per week in weeks 1–4, with two sets of each exercise and each set consisting of 8–12 repetitions and then three times per week in weeks 5–8, with three sets of each exercise (each set includes 8–12 repetitions), with plenty of rest between sets[27-30].

For peer review only - http://bmjopen.bmj.com/site/about/guidelines.xhtml

Hip abduction: This exercise is done in a standing position, with the elastic band around the ankle of one foot while the other foot is on the elastic band. The opposite hand holds the seat, and the supporting foot is firmly planted on the ground to keep the trunk stable. Initially, the buttocks exert force as the elastic band is stretched outward to the side by raising the leg until the angle between the practice leg and the supporting foot is about 30 degrees. After a pause for about 1 second, the leg is slowly lowered to the ground. The exercise is done by alternating the left and right feet between sets.

Seated knee raising: This exercise is done in a sitting position with a straight back while holding the seat with both hands, keeping the upper torso upright, stepping on one end of the elastic band with one foot, and pulling the elastic band around the ankle of the other foot. The knee is then lifted to the maximum angle of joint activity and the after a pause for about 1 second, slowly lowered. The exercise is done by alternating the left and right legs between sets.

Raise before standing: While standing with feet parallel, head straight and looking straight ahead, the dumbbells are held at both sides of the body by both hands. Both arms are then raised in front of the body, and after a pause for about 1 second, the arms are slowly lowered. Participants are instructed to inhale during the horizontal lift and exhale while returning the arms to their original positions.

Standing lateral raise: While standing with feet parallel, head straight and looking straight ahead, the dumbbells are held at both sides of the body by both hands. The arms are raised from the sides, with elbows slightly bent and focus placed on the shoulders. Once the arm is raised to a horizontal position, participants pause for about 1 second before slowly lowering the arms to the initial positions. Participants are instructed to inhale during the horizontal lift and exhale while returning the arms to their original positions.

The intensity of aerobic exercise should be moderate; that is, the Borg subjective fatigue scale score should reach 13-–14 points (feeling a little hard). The intensity of resistance exercise should be tolerable to the patient. If obvious fatigue and muscle pain occur during or after training, the intensity of physical activity should be appropriately reduced. If patients have severe pain, discomfort and other symptoms, they should immediately stop the activity and be admitted to the hospital for re-examination[31,32].

During the exercise, family members are instructed to pay attention to the patient’s reactions and to adjust the elastic band or exercise speed and intensity as needed. They must also ensure the patient is not straining or using too much force during exercise to prevent stretching the wound or the place where the catheter is placed. They should also pay attention to the patient’s movement at all times. If the patient experiences dizziness, discomfort or other adverse events, they should be promptly instructed to stop exercising and visit the hospital if necessary.

Outcome measurements

For peer review only - http://bmjopen.bmj.com/site/about/guidelines.xhtml

The outcome measurements for this pilot study will be derived from baseline assessments and clinical outcomes.

Demographic and clinical characteristics of the participants

A self-designed demographic and clinical data form will be employed to collect the participants' sociodemographic data (e.g., age, educational background, employment status, marital status, and household income), the participants' medical history (e.g., date of BC diagnosis, current stage of BC, and date and type of treatment) and family members' sociodemographic data at baseline (T0).

Primary outcome: CRF

Piper Fatigue Scale-Revised (R-PFS)[33]: The R-PFS will be applied to assess the participants' subjective fatigue. This self-administered questionnaire contains 22 items with scores ranging from 0 to 10 and includes four domains of subjective fatigue. The R-PFS is available in a simplified Chinese version[34], with high reliability and validity, and has high reliability in the BC population.

Secondary outcomes: muscle power, exercise completion, family intimacy and adaptability. and quality of life

Muscle strength, exercise completion, family intimacy and adaptability, and patient quality of life will be assessed as secondary outcomes at T0, T1, and T2 using the stand-up and sit-down chair test, grip test, exercise completion rate, FACES Ⅱ -CV and FACT-B.

Stand-up and sit-down chair test (number of times standing up from the chair within 30 seconds) [35]: This test will be used to evaluate the leg strength of the participant. The test procedure is as follows: 1) an upright chair (or a folding chair) is placed against a wall (for the sake of safety); 2) the participant sit in the middle of the chair with the right and left feet shoulder-width apart, and one foot may be put slightly forward and the other slightly back, while both arms are crossed at the waist and held near the chest; 3) during the test, participants should completely stand up and then completely sit down; 4) the number of times that the participant stands up from and sits down in the chair within 30 seconds is recorded; and 5) for safety purposes or when necessary, the participant may use his or her arms for assistance.

Grip test[32]: This test will be used to evaluate the arm strength of the participants. A CAMRY (model EH101) electronic grip strength meter is used in units of kg/lb. The maximum force is 90 kg/198 lb, and the division value is 0.1 kg/0.2 lb. Participants are in a standing position, with their hands hanging down naturally, while their feet are positioned under the shoulders and hands are held away from the body.

Exercise completion rate: This test will be used to assess the performance of the two types of exercise. The exercise completion rate in this study will be calculated by dividing the number of participants in the intervention group who completed 18 or more training sessions (more than 90% of the total training volume) by the total number of people in the intervention group and multiplying that value by 100%.

For peer review only - http://bmjopen.bmj.com/site/about/guidelines.xhtml

FACESⅡ-CV[36]: The FACESⅡ-CV will be adopted to assess the participants' family intimacy and adaptability. A higher score indicates higher intimacy and adaptability. The FACESⅡ-CV is available in a simplified Chinese version, with high reliability and validity.

FACT-B[37]: The FACT-B will be adopted to assess the patients' quality of life. A higher score reflects better quality of life. The FACT-B is available in a simplified Chinese version, with adequate psychometric properties reported among patients with BC.

Data analysis

Statistical analysis of the results will be completed using SPSS software version 25.0. Analysis of covariance will be applied for comparisons between groups, and paired t-tests will be used for comparisons from before to after exercise within a group. Values of $p<0.05$ will represent that a significant difference has been detected between test results.

Data management

After data collection is complete, all handwritten data will be converted to electronic data. All data will be independently recorded by two researchers in Excel spreadsheets. The software will automatically check for inconsistent or problematic data based on the inspection results and generate a data problem table. After all data have been confirmed, reconciled, and stored in an electronic database, participants' identifying information (e.g., real name) will not appear in the relevant reports of the trial to protect their privacy. Only researchers directly involved in the analysis of this study will have access to the final trial dataset, which will contain only coded data.

Data sharing statement

A technical appendix, statistical codes and dataset will be available at any time upon request to the corresponding author.

Patient and public involvement

Patients or the public were not involved in the design of this study and will not be involved in conducting the research or reporting the results.

Ethics and dissemination

Ethical approval of the proposed study has been granted by the Ethics Committee of the First Affiliated Hospital of Dalian Medical University (PJ-KS-KY-2021-288), and the trial has been registered in the Chinese Clinical Trial Registry (registration number: ChiCTR2200055793). Only participants who provided written informed consent will be included in the study. The results of this study will be published via peer-reviewed publications and presentations at conferences.

DISCUSSION

For peer review only

For peer review only - http://bmjopen.bmj.com/site/about/guidelines.xhtml

As one of the most common symptom clusters in patients with BC, CRF can significantly diminish patients' quality of life and daily functioning[38]. An increasing number of studies has demonstrated that combined aerobic and resistance exercise has beneficial effects on symptom management in patients with cancer[39]. However, due to differences in the time and intensity of exercise as well as individual differences, the effect of such exercise has varied among different studies. Gokal et al.[40] tested a home-based walking intervention based on self-management in 25 BC patients receiving chemotherapy and found that this self-management exercise intervention could improve patients' fatigue symptoms as well as their physical activity level. However, some patients had difficulty adhering to exercise training, causing them to stop exercising. Uhm et al.[41] conducted a 12-week RCT on 356 BC patients, which showed that combined aerobic and resistance exercise improved patients' fatigue symptoms, muscle strength, and quality of life. However, because some patients could not persist in completing the exercise, the advantages of the tested exercise over traditional exercise programs were not obvious. Previous studies have confirmed that family-involvement exercise interventions have many benefits for patients after stroke[42], coronary heart disease[43], lung cancer[44], and other conditions, but there are few reports on the effect of family-involvement exercise interventions in BC patients in China. This highlights a great need to explore the effects of family-involvement exercise on CRF in patients with BC.

The principal strength (and novel aspect) of this study resides in the fact that in the intervention, each participant will be able to choose the type of aerobic exercise they prefer. Also, the study will provide exercise instruction videos designed to increase patients' motivation to exercise. In addition, we aim to provide new evidence on the cost-effectiveness of this type of intervention (to our knowledge, virtually no evidence has previously been reported in this regard). Moreover, an exercise booklet will be provided to the participants in both the intervention and control groups. The information presented in this booklet has been comprehensively adapted from relevant national guidelines, professional bodies, and research evidence in published peer-reviewed articles.

The proposed study will also have some limitations. Given the limited number of study sites, the study sample may not offer a widely representative sample of patients with BC. Moreover, due to the visible nature of the exercise intervention, blinding of the participants and investigators cannot be performed in this study, which might increase the risk of detection bias during the study's implementation. The lack of long-term follow-up to assess the ongoing effects of family-involvement exercise might be another limitation, but this can be considered in the future full-scale trial as one of the main study outcomes. Furthermore, this study will not be a rigorous RCT but rather a Q-RCT, and a future multi-center, large-scale RCT will need to be conducted to further verify the research evidence for the effects of family-involvement exercise on the management of BC patients with CRF.

For peer review only - http://bmjopen.bmj.com/site/about/guidelines.xhtml

The proposed Q-RCT will assess the preliminary effects of a family-involvement exercise program for alleviating CRF in BC patients undergoing postoperative chemotherapy. The convenience of the family-involvement exercise for the management of CRF may provide patients, healthcare professionals, and policy-makers with further guidance for CRF management in the long-term.

Author affiliations

[1]Department of Nursing, The First Affiliated Hospital of Dalian Medical University, Dalian, China

Contributorship statement: Chuhan Huang conceived and designed the study, and Tieying Shi will oversee the research team and research process. Yingjie Cai and Chuhan Huang will be the main implementers of the study and drafted this manuscript. Jingjing Jia wrote the ethics review confirmation. Yufei Guo participated in the design of the study and assisted in drafting this manuscript. All authors have read and agreed to the final version of the manuscript.

Funding: Preparation of this study protocol was funded by the Dalian Science and Technology Innovation Fund Science and Technology Benefit People Project (Award/Grant No. 2022JJ13FG109). The authors have not declared a specific grant that will fund the proposed research from any funding agency in the public, commercial or not-for-profit sectors.

Competing interests: None declared.

Patient consent for publication: Not required for present manuscript.

Provenance and peer review: Not commissioned; externally peer reviewed.

Ethics approval: The study protocol has been approved by the Ethics Committee of the First Affiliated Hospital of Dalian Medical University, China (PJ-KS-KY-2021-288).

REFERENCES

[1]Sung H,Global cancer statistics 2020: GLOBOCAN estimates of incidence and mortality world wide for 36 cancers in 185 countries[J]. A Cancer Journal for Clinicians. 2021, 71(3): 209-249

[2]World Health Organization. Global Cancer Observatory(GCO):Cancer Today[EB/OL] https://gcoiarcfr/today/home, 2021-02-03.

[3]Chen W, Zheng R, Baade PD, et al. Cancer statistics in China, 2015[J]. A Cancer Journal for Clinicians. 2016 ,66(2):115-32.

[4]Jiang Xiaoting, Liu Qiang, Song Erwei. Questions and reflections in the diagnosis and treatment of younger breast cancer in China[J]. National Medical Journal of China, 2022, 102(36): 2823-2827.

[5]Li T, Mello-Thoms C, Brennan PC. Descriptive epidemiology of breast cancer in China: incidence, mortality, survival and prevalence[J]. Breast Cancer Research and Treatment, 2016, 159(3): 395-406.

[6]Breast Cancer Professional Committee of China Anti-Cancer Association. Chinese Anti-Cancer Association Guidelines and Standards for Breast Cancer Diagnosis and Treatment (2021

For peer review only - http://bmjopen.bmj.com/site/about/guidelines.xhtml

edition)[J].China Oncology, 2021, 31(10): 954-1040.
[7]Peng Z, Wei J, Lu X, et al.Diagnosis and treatment pattern among rural and urban breast cancer patients in Southwest China from 2005 to 2009[J]. Oncotarget. 2016 , 7(47): 78168-78179.
[8]Sue Kim, Jeehee Han, Min Young Lee, et al. The experience of cancer-related fatigue, exercise and exercise adherence among women breast cancer survivors: Insights from focus group interviews[J]. Journal of Clinical Nursing, 2020, 29(5-6): 758-769.
[9]Fabi A, Bhargava R, Fatigoni S, et al. Cancer-related Fatigue: Esmo Clinical Practice Guidelines for Diagnosis and Treatment[J]. Annals of Oncology, 2020, 31(6): 713-723.
[10]Abrahams HJG, Gielissen MFM, Schmits IC, et al. Risk factors, prevalence, and course of severe fatigue after breast cancer treatment: a meta-analysis involving 12 327 breast cancer survivors[J]. Annals of Oncology, 2016, 27(6): 965-974.
[11]Ekenga CC, Perez M, Margenthaler JA, et al. Early-stage Breast Cancer and Employment Participation after 2 Years of Follow-up: A Comparison with Age-matched Controls[J]. Cancer, 2018, 124(9): 2026-2035.
[12]Mustian KM, Alfano CM, Heckler CE, et al. Comparison of pharmaceutical, psychological, and exercise treatments for cancer-related fatigue: a meta-analysis[J]. JAMA Oncology, 2017, 3(7):961-968.
[13]Ehlers Diane K, DuBois Kelli, Salerno Elizabeth A. The effects of exercise on cancer-related fatigue in breast cancer patients during primary treatment: a meta-analysis and systematic review[J]. Expert Review of Anticancer Therapy, 2020, 20(10): 865-877.
[14]Lahart Ian M, Metsios George S, Nevill Alan M, et al. Physical activity for women with breast cancer after adjuvant therapy[J]. The Cochrane database of systematic reviews, 2018, 1(1): CD011292.
[15]Caspersen CJ, Powell KE, Christenson GM. Physical activity, exercise, and physical fitness: definitions and distinctions for health-related research[J]. Public Health Reports, 1985, 100(2): 126-131.
[16]Breast Health Group(BEST: Breast Education Screening Diagnosis and Treatment Group) of the Branch of Women Health of Chinese Preventive Medicine Association. Guidelines on life-style modification for Chinese breast cancer surviors[J]. Chinese Journal of Surgery, 2017, 55(2): 81-85.
[17]Joel T Fuller, Michael C Hartland, Luke T Maloney, et al. Therapeutic effects of aerobic and resistance exercises for cancer survivors: a systematic review of meta-analyses of clinical trials[J]. British Journal of Sports Medicine,2018, 52(20): 1311.
[18]ZHOU Shuai, JIANG Jinfang, ZHANG Ling, et al. Different Exercise Therapies in Patients with Cancer-related Fatigue: A Network Meta-Analysis[J].Nursing Journal of Chinese People's Liberation Army, 2021, 38(08): 65-68+88.
[19]DENG Yangqing, PAN Cen, WANG Qinglan,et al.Qualitative Research on Exercise Experience of Patients with Breast Cancer: A Meta-synthesis[J].Journal of Nursing(China), 2021, 28(13): 34-39.
[20]Liu Min, Li Guohong, Xue Xiaoming, et al. Effect of family-participated continuation nursing model on the quality of life of discharged patients with colorectal cancer[J].Chinese Journal of Gerontology, 2020, 40(23): 5080-5085.
[21]Wanderson Divino Nilo dos Santos, Amilton Vieira, Claudio Andre Barbosa de Lira, et al. Once a Week Resistance Training Improves Muscular Strength in Breast Cancer Survivors: A Randomized Controlled Trial[J]. Integrative Cancer Therapies,2019,18, 1534735419879748..
[22]Witlox L, Velthuis MJ, Boer JH, et al. Attendance and compliance with an exercise program

For peer review only

For peer review only - http://bmjopen.bmj.com/site/about/guidelines.xhtml

during localized breast cancer treatment in a randomized controlled trial: The PACT study[J]. PLoS One,2019, 14(5): e0215517.
[23]YUN Qingping, JI Ying, JIANG Xuewen, et al.Path analysis of health behavior improvement in chronic disease patients based on family function theory[J].Chinese Journal of Disease Control & Prevention, 2019, 23(07): 861-865.
[24]HE Peishan, PAN Guofeng, YANG Gongbo, et al. Observation on Acupoint Application with "Wenyang Yiqi Formula" Treating Cancer-Related Fatigue on Breast Cancer Patients with Chemotherapy[J]. Lishizhen Medicine and Materia Medica Research, 2019, 30(2): 391-393.
[25]Jiang Yongqin, Yan Ling, Liu Chunyan, et al. Effects of exercise prescription on cancer-related fatigue in breast cancer patients[J]. Chinese Journal of Nursing, 2008(10): 906-909.
[26]Yan Ling. Effects of exercise prescription on cancer-related fatigue in breast cancer patients[D].Tianjin Medical University, 2008.
[27]Anne Marthe Schreuder, Anne M. Eskes, Rosanna G.M. van Langen, et al. Active involvement of family members in postoperative care after esophageal or pancreatic resection: A feasibility study[J]. Surgery, 2019, 166(5): 769-777.
[28]Huang HP, Wen FH, Yang TY, et al. The effect of a 12-week home-based walking program on reducing fatigue in women with breast cancer undergoing chemotherapy: A randomized controlled study[J]. International Journal of Nursing Studies, 2019, 11(9): 103376.
[29]Li He, Wang Yixiao, Shi Tieying,et al. Effect of Progressive Resistance Training on Upper Limb Function in Breast Cancer Patients[J].Chinese Journal of Rehabilitation Theory and Practice, 2015, 21(11): 1356-1359.
[30]Qin Bo. Effects of elastic band resistance training on lower limb muscle strength and physical activity in the elderly[D].Shanghai Normal University, 2021.
[31]Dong Xin, Mo Yihan, WANG Xiuhua. Effects of resistance exercise on muscle mass, muscle strength and physical performance in the elderly at risk of sarcopenia[J].Chinese Nursing Management, 2021, 21(08): 1190-1195.
[32]Li He. Postoperative progressive resistance training on the rehabilitation of breast cancer patients[D]. Dalian Medical University, 2015.
[33]So WKW, Dodgson J, Tai JWM. Fatigue and quality of life among Chinese patients with hematologic malignancy after bone marrow transplantation[J]. Cancer Nursing, 2003, 26(3):211-219.
[34]Li L, Zhu LH, Cao MQ. Progress in the diagnosis and evaluation scale of cancer-related fatigue[J]. Xinjiang Medcine Journal, 2016, 46(11): 1458-1461,1465.
[35]Xiaosheng Dong, Xiangren Yi, Dezong Gao, et al. The effects of the combined exercise intervention based on internet and social media software (CEIBISMS) on quality of life, muscle strength and cardiorespiratory capacity in Chinese postoperative breast cancer patients:a randomized controlled trial[J]. BioMed Central,2019,17(1): 109.
[36]Fei Lipeng, Shen Qijie, Zheng Yanping, et al. Preliminary evaluation of "Family Intimacy and Adaptability Scale" and "Family Environment Scale"[J].Chinese Mental Health Journal, 1991(05): 198-202+238.
[37]Wan Chonghua, Tang Xueliang. Introduction to the Chinese version of FACT-B, a measure of quality of life for breast cancer patients[J]. China Cancer, 2002, 11(6):318-320.
[38]Ruiz-Casado A, Álvarez-Bustos A, de Pedro CG, et al. Cancer-related Fatigue in Breast Cancer Survivors: A Review[J]. Clinical Breast Cancer. 2021, 21(1): 10-25.
[39]BOLAM K A, MIJWEL S, RUNDQVIST H, et al. Two-year follow-up of the OptiTrain randomised controlled exercise trial[J]. Breast Cancer Research and Treatment, 2019, 175(3): 637-

For peer review only

648.
[40]Gokal Kajal, Wallis Deborah, Ahmed Samreen, et al. Effects of a self-managed home-based walking intervention on psychosocial health outcomes for breast cancer patients receiving chemotherapy: a randomised controlled trial[J]. Supportive care in cancer, 2016, 24(3): 1139-66..
[41]Kyeong Eun Uhm, Ji Sung Yoo, Seung Hyun Chung, et al. Effects of exercise intervention in breast cancer patients: is mobile health (mHealth) with pedometer more effective than conventional program using brochure[J]. Breast Cancer Research and Treatment,2017,161(3):443-452.
[42]Galvin R, Cusack T, Stokes E. A randomised controlled trial evaluating family mediated exercise (FAME) therapy following stroke[J]. BMC Neurology. 2008, 20(8): 22.
[43]Ma Lili, Sun Suping, YAN Xueqing, et al. Effects of cardiac rehabilitation with the participation of spouses on the patients undergoing PCI and their spouses[J].Chinese Nursing Management, 2020, 20(03): 393-398.
[44]Kelly J. Lafaro, Dan J. Raz, Jae Y. Kim, et al. Pilot study of a telehealth perioperative physical activity intervention for older adults with cancer and their caregivers[J]. Supportive Care in Cancer,2020,28(8): 3867-3876.

Figure 1 A Consolidated Standards of Reporting Trials flow chart of the study

For peer review only

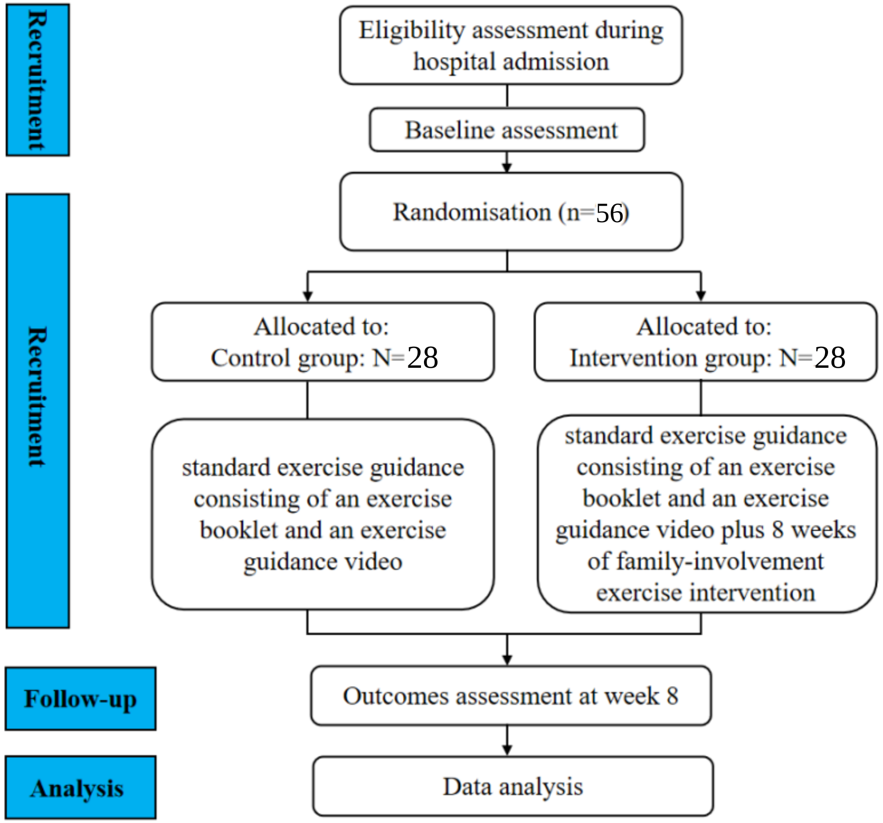
Recruitment
Eligibility assessment during hospital admission
Baseline assessment
Recruitment
Randomisation (n=56)
Allocated to:
Control group: N=28
Allocated to:
Intervention group: N=28
standard exercise guidance consisting of an exercise booklet and an exercise guidance video
standard exercise guidance consisting of an exercise booklet and an exercise guidance video plus 8 weeks of family-involvement exercise intervention
Follow-up
Outcomes assessment at week 8
Analysis
Data analysis

STANDARD PROTOCOL ITEMS: RECOMMENDATIONS FOR INTERVENTIONAL TRIALS

SPIRIT 2013 Checklist: Recommended items to address in a clinical trial protocol and related documents*

Section/item	Item No	Description	Reported on page No
**Administrative information**			
Title	1	Descriptive title identifying the study design, population, interventions, and, if applicable, trial acronym	Effect of a family-involvement combined aerobic and resistance exercise protocol on cancer-related fatigue in breast cancer patients during postoperative chemotherapy:<br>study protocol for a quasi-randomized controlled trial<br>(No.1/No.2)
Trial registration	2a	Trial identifier and registry name. If not yet registered, name of intended registry	ChiCTR2200055793(No.12)
	2b	All items from the World Health Organization Trial Registration Data Set	The trial has been registered in the Chinese Clinical Trial Registry(No.12)
Protocol version	3	Date and version identifier	Date: 2021.10.12<br>Version 1.0
Funding	4	Sources and types of financial, material, and other support	Preparation of this study protocol was funded by the Dalian Science and Technology Innovation Fund Science and Technology Benefit People Project (Award/Grant No. 2022JJ13FG109).(No.13)
Roles and responsibilities	5a	Names, affiliations, and roles of protocol contributors	Names:<br>Chuhan Huang<br>Affiliations:<br>The First Affiliated Hospital of Dalian Medical University<br>Roles:<br>The general design of the protocol
	5b	Name and contact information for the trial sponsor	The study has not any trial sponsor.

For peer review only - http://bmjopen.bmj.com/site/about/guidelines.xhtml

	5c	Role of study sponsor and funders, if any, in study design; collection, management, analysis, and interpretation of data; writing of the report; and the decision to submit the report for publication, including whether they will have ultimate authority over any of these activities	The study has not any trial sponsor.
	5d	Composition, roles, and responsibilities of the coordinating centre, steering committee, endpoint adjudication committee, data management team, and other individuals or groups overseeing the trial, if applicable (see Item 21a for data monitoring committee)	It has not yet been identified the Composition of data monitoring committee (DMC) in the study protocol.
**Introduction**			
Background and rationale	6a	Description of research question and justification for undertaking the trial, including summary of relevant studies (published and unpublished) examining benefits and harms for each intervention	It has been elucidated in the study protocol context.(No.3-4)
	6b	Explanation for choice of comparators	The researcher will provide exercise guidance for “combined aerobic and resistance exercise”for the control group to verify the effect of family members’ participation (No.6-7)
Objectives	7	Specific objectives or hypotheses	The current study, therefore, proposes to assess the preliminary effects of a family-involvement exercise protocol on alleviating CRF in BC patients through a quasi-randomized controlled trial (Q-RCT).(No.4)
Trial design	8	Description of trial design including type of trial (eg, parallel group, crossover, factorial, single group), allocation ratio, and framework (eg, superiority, equivalence, noninferiority, exploratory)	The trial will be a quasi-randomized controlled trial (Q-RCT).(No.4)
**Methods: Participants, interventions, and outcomes**			

For peer review only

Study setting	9	Description of study settings (eg, community clinic, academic hospital) and list of countries where data will be collected. Reference to where list of study sites can be obtained	Patients receiving postoperative chemotherapy for BC in a Grade A tertiary hospital in Dalian will be selected for this study.(No.4)
Eligibility criteria	10	Inclusion and exclusion criteria for participants. If applicable, eligibility criteria for study centres and individuals who will perform the interventions (eg, surgeons, psychotherapists)	It is already described in the part "Participant Recruitment and Eligibility Criteria" of the main docment.(No.4-5)
Interventions	11a	Interventions for each group with sufficient detail to allow replication, including how and when they will be administered	The text has been described at page 5-10.
	11b	Criteria for discontinuing or modifying allocated interventions for a given trial participant (eg, drug dose change in response to harms, participant request, or improving/worsening disease)	The text has been described at page 9-10.
	11c	Strategies to improve adherence to intervention protocols, and any procedures for monitoring adherence (eg, drug tablet return, laboratory tests)	The text has been described at page 7.
	11d	Relevant concomitant care and interventions that are permitted or prohibited during the trial	Relevant concomitant care and interventions that are not prohibited during the trial.
Outcomes	12	Primary, secondary, and other outcomes, including the specific measurement variable (eg, systolic blood pressure), analysis metric (eg, change from baseline, final value, time to event), method of aggregation (eg, median, proportion), and time point for each outcome. Explanation of the clinical relevance of chosen efficacy and harm outcomes is strongly recommended	The text has been described at page 10.

For peer review only - http://bmjopen.bmj.com/site/about/guidelines.xhtml

Participant timeline	13	Time schedule of enrolment, interventions (including any run-ins and washouts), assessments, and visits for participants. A schematic diagram is highly recommended (see Figure)	The text has been described at page 6, figure 1.
Sample size	14	Estimated number of participants needed to achieve study objectives and how it was determined, including clinical and statistical assumptions supporting any sample size calculations	The text has been described at page 5.
Recruitment	15	Strategies for achieving adequate participant enrolment to reach target sample size	Due to the sufficient sample size in this study, the strategies for achieving adequate participant enrolment to reach target sample size will be not adopted.
**Methods: Assignment of interventions (for controlled trials)**			
Allocation:			
Sequence generation	16a	Method of generating the allocation sequence (eg, computer-generated random numbers), and list of any factors for stratification. To reduce predictability of a random sequence, details of any planned restriction (eg, blocking) should be provided in a separate document that is unavailable to those who enrol participants or assign interventions	This study used a quasi-randomized controlled trial without assignment of sequence generation.
Allocation concealment mechanism	16b	Mechanism of implementing the allocation sequence (eg, central telephone; sequentially numbered, opaque, sealed envelopes), describing any steps to conceal the sequence until interventions are assigned	This study has not allocation concealment mechanism.
Implementation	16c	Who will generate the allocation sequence, who will enrol participants, and who will assign participants to interventions	The text has been described at page 5.

Blinding (masking)	17a	Who will be blinded after assignment to interventions (eg, trial participants, care providers, outcome assessors, data analysts), and how	No blinding was set in this study due to the limited study conditions.
	17b	If blinded, circumstances under which unblinding is permissible, and procedure for revealing a participant's allocated intervention during the trial	No blinding was set in this study due to the limited study conditions.
**Methods: Data collection, management, and analysis**			
Data collection methods	18a	Plans for assessment and collection of outcome, baseline, and other trial data, including any related processes to promote data quality (eg, duplicate measurements, training of assessors) and a description of study instruments (eg, questionnaires, laboratory tests) along with their reliability and validity, if known. Reference to where data collection forms can be found, if not in the protocol	The text has been described at page 5, 9-11.
	18b	Plans to promote participant retention and complete follow-up, including list of any outcome data to be collected for participants who discontinue or deviate from intervention protocols	The text has been described at page 11.
Data management	19	Plans for data entry, coding, security, and storage, including any related processes to promote data quality (eg, double data entry; range checks for data values). Reference to where details of data management procedures can be found, if not in the protocol	The text has been described at page 11.
Statistical methods	20a	Statistical methods for analysing primary and secondary outcomes. Reference to where other details of the statistical analysis plan can be found, if not in the protocol	The text has been described at page 11.
	20b	Methods for any additional analyses (eg, subgroup and adjusted analyses)	This study has not any methods for any additional analyses.

For peer review only - http://bmjopen.bmj.com/site/about/guidelines.xhtml

	20c	Definition of analysis population relating to protocol non-adherence (eg, as randomised analysis), and any statistical methods to handle missing data (eg, multiple imputation)	It is not involved definition of analysis population relating to protocol non-adherence and any statistical methods to handle missing data.
**Methods: Monitoring**			
Data monitoring	21a	Composition of data monitoring committee (DMC); summary of its role and reporting structure; statement of whether it is independent from the sponsor and competing interests; and reference to where further details about its charter can be found, if not in the protocol. Alternatively, an explanation of why a DMC is not needed	It has not yet been identified the Composition of data monitoring committee (DMC) in the study protocol. The research team will monitor and manage the data.
	21b	Description of any interim analyses and stopping guidelines, including who will have access to these interim results and make the final decision to terminate the trial	Graduate students in the research group will conduct any interim analyses.
Harms	22	Plans for collecting, assessing, reporting, and managing solicited and spontaneously reported adverse events and other unintended effects of trial interventions or trial conduct	The text has been described at page 9-10.
Auditing	23	Frequency and procedures for auditing trial conduct, if any, and whether the process will be independent from investigators and the sponsor	The process which auditing trial conduct will be independent from investigators.
**Ethics and dissemination**			
Research ethics approval	24	Plans for seeking research ethics committee/institutional review board (REC/IRB) approval	The text has been described at page 11-12.
Protocol amendments	25	Plans for communicating important protocol modifications (eg, changes to eligibility criteria, outcomes, analyses) to relevant parties (eg, investigators, REC/IRBs, trial participants, trial registries, journals, regulators)	The study protocol has been communicated with the Ethics Committee of the First Affiliated Hospital of Dalian Medical University.

For peer review only - http://bmjopen.bmj.com/site/about/guidelines.xhtml

Consent or assent	26a	Who will obtain informed consent or assent from potential trial participants or authorised surrogates, and how (see Item 32)	The text has been described at page 12.
	26b	Additional consent provisions for collection and use of participant data and biological specimens in ancillary studies, if applicable	It is not involved additional consent provisions for collection and use of participant data and biological specimens in ancillary studies.
Confidentiality	27	How personal information about potential and enrolled participants will be collected, shared, and maintained in order to protect confidentiality before, during, and after the trial	This process will be all performed by one investigator.
Declaration of interests	28	Financial and other competing interests for principal investigators for the overall trial and each study site	The text has been described at page 13.
Access to data	29	Statement of who will have access to the final trial dataset, and disclosure of contractual agreements that limit such access for investigators	The text has been described at page 11.
Ancillary and post-trial care	30	Provisions, if any, for ancillary and post-trial care, and for compensation to those who suffer harm from trial participation	It is not involved ancillary and post-trial care.
Dissemination policy	31a	Plans for investigators and sponsor to communicate trial results to participants, healthcare professionals, the public, and other relevant groups (eg, via publication, reporting in results databases, or other data sharing arrangements), including any publication restrictions	The text has been described at page 13.
	31b	Authorship eligibility guidelines and any intended use of professional writers	The text has been described at page 13.
	31c	Plans, if any, for granting public access to the full protocol, participant-level dataset, and statistical code	The text has been described at page 11.

Appendices

For peer review only - http://bmjopen.bmj.com/site/about/guidelines.xhtml

Informed consent materials	32	Model consent form and other related documentation given to participants and authorised surrogates	Model consent form and other related documentation will be given to participants and authorised surrogates.
Biological specimens	33	Plans for collection, laboratory evaluation, and storage of biological specimens for genetic or molecular analysis in the current trial and for future use in ancillary studies, if applicable	Biological specimens will be not involved in this study.

*It is strongly recommended that this checklist be read in conjunction with the SPIRIT 2013 Explanation & Elaboration for important clarification on the items. Amendments to the protocol should be tracked and dated. The SPIRIT checklist is copyrighted by the SPIRIT Group under the Creative Commons "Attribution-NonCommercial-NoDerivs 3.0 Unported" license.

For peer review only
